# Past and future spread of the arbovirus vectors *Aedes aegypti* and *Aedes albopictus*

**DOI:** 10.1038/s41564-019-0376-y

**Published:** 2019-03-04

**Authors:** Moritz U. G. Kraemer, Robert C. Reiner, Oliver J. Brady, Jane P. Messina, Marius Gilbert, David M. Pigott, Dingdong Yi, Kimberly Johnson, Lucas Earl, Laurie B. Marczak, Shreya Shirude, Nicole Davis Weaver, Donal Bisanzio, T. Alex Perkins, Shengjie Lai, Xin Lu, Peter Jones, Giovanini E. Coelho, Roberta G. Carvalho, Wim Van Bortel, Cedric Marsboom, Guy Hendrickx, Francis Schaffner, Chester G. Moore, Heinrich H. Nax, Linus Bengtsson, Erik Wetter, Andrew J. Tatem, John S. Brownstein, David L. Smith, Louis Lambrechts, Simon Cauchemez, Catherine Linard, Nuno R. Faria, Oliver G. Pybus, Thomas W. Scott, Qiyong Liu, Hongjie Yu, G. R. William Wint, Simon I. Hay, Nick Golding

**Affiliations:** 1 Department of Zoology, University of Oxford, Oxford, UK; 2 Harvard Medical School, Harvard University, Boston, MA USA; 3 Boston Children’s Hospital, Boston, MA USA; 4 Institute for Health Metrics and Evaluation, University of Washington, Seattle, WA USA; 5 Centre for Mathematical Modelling of Infectious Diseases, London School of Hygiene and Tropical Medicine, London, UK; 6 Department of Infectious Disease Epidemiology, London School of Hygiene and Tropical Medicine, London, UK; 7 School of Geography and the Environment, University of Oxford, Oxford, UK; 8 Oxford School of Global and Area Studies, University of Oxford, Oxford, UK; 9 Spatial Epidemiology Lab (SpELL), Universite Libre de Bruxelles, Brussels, Belgium; 10 Fonds National de la Recherche Scientifique, Brussels, Belgium; 11 Department of Statistics, Harvard University, Cambridge, MA USA; 12 RTI International, Washington, DC USA; 13 Epidemiology and Public Health Division, School of Medicine, University of Nottingham, Nottingham, UK; 14 Department of Biological Sciences and Eck Institute for Global Health, University of Notre Dame, Notre Dame, IN USA; 15 School of Health, Fudan University, Key Laboratory of Public Health Safety, Ministry of Education, Shanghai, China; 16 Department of Geography and Environment, University of Southampton, Southampton, UK; 17 Flowminder Foundation, Stockholm, Sweden; 18 School of Business, Central South University, Changsha, China; 19 College of Systems Engineering, National University of Defense Technology, Changsha, China; 20 School of Business Administration, Southwestern University of Finance and Economics, Chengdu, China; 21 Waen Associates Ltd, Y Waen, Islaw’r Dref, Dolgellau, Gwynedd, UK; 22 Pan American Health Organization (PAHO), Washington, DC USA; 23 National Dengue Control Program, Ministry of Health, Brasilia, Brazil; 24 European Centre for Disease Prevention and Control, Stockholm, Sweden; 25 Institute of Tropical Medicine, Antwerp, Belgium; 26 Avia-GIS, Zoersel, Belgium; 27 Francis Schaffner Consultancy, Riehen, Switzerland; 28 Department of Microbiology, Immunology, and Pathology, Colorado State University, Fort Collins, CO USA; 29 Computational Social Science, ETH Zurich, Zurich, Switzerland; 30 Department of Public Health Sciences, Karolinska Institutet, Stockholm, Sweden; 31 Stockholm School of Economics, Stockholm, Sweden; 32 Insect–Virus Interactions Unit, Institut Pasteur, CNRS, UMR2000 Paris, France; 33 Mathematical Modelling of Infectious Diseases Unit, Institut Pasteur, CNRS, UMR2000 Paris, France; 34 Department of Geography, Universite de Namur, Namur, Belgium; 35 Department of Entomology and Nematology, University of California, Davis, Davis, CA USA; 36 State Key Laboratory of Infectious Disease Prevention and Control, Collaborative Innovation Center for Diagnosis and Treatment of Infectious Diseases, National Institute for Communicable Disease Control and Prevention, Chinese Center for Disease Control and Prevention, Changping, Beijing, China; 37 Shandong University Climate Change and Health Center, School of Public Health, Shandong University, Jinan, Shandong, China; 38 WHO Collaborating Centre for Vector Surveillance and Management, Beijing, China; 39 Chongqing Centre for Disease Control and Prevention, Chongqing, China; 40 Environmental Research Group Oxford (ERGO), Department of Zoology, Oxford University, Oxford, UK; 41 School of BioSciences, University of Melbourne, Parkville, Victoria Australia

**Keywords:** Microbiology, Infectious diseases

## Abstract

The global population at risk from mosquito-borne diseases—including dengue, yellow fever, chikungunya and Zika—is expanding in concert with changes in the distribution of two key vectors: *Aedes aegypti* and *Aedes albopictus*. The distribution of these species is largely driven by both human movement and the presence of suitable climate. Using statistical mapping techniques, we show that human movement patterns explain the spread of both species in Europe and the United States following their introduction. We find that the spread of *Ae. aegypti* is characterized by long distance importations, while *Ae. albopictus* has expanded more along the fringes of its distribution. We describe these processes and predict the future distributions of both species in response to accelerating urbanization, connectivity and climate change. Global surveillance and control efforts that aim to mitigate the spread of chikungunya, dengue, yellow fever and Zika viruses must consider the so far unabated spread of these mosquitos. Our maps and predictions offer an opportunity to strategically target surveillance and control programmes and thereby augment efforts to reduce arbovirus burden in human populations globally.

## Abstract (web summary)

Statistical mapping techniques provide insights into the spread of two key arbovirus vectors in Europe and the United States, and predict the future distributions of both mosquitoes in response to accelerating urbanization, connectivity and climate change.

## Main

The geographical distributions of the arboviruses dengue, yellow fever, chikungunya and Zika have expanded, causing severe disease outbreaks in many urban populations^1–5^. Transmission of these viruses depends, with few exceptions, on the presence of the competent mosquito vectors *Ae*. *aegypti* (also known as *Stegomyia aegypti*) and *Ae. albopictus* (also known as *Stegomyia albopicta*)^6,7^. Previous predictions of the future distributions of *Ae. aegypti* and *Ae. albopictus* have focused solely on climate, despite the known importance of urbanization and other socioeconomic factors in defining suitable habitat^8^. Moreover, those projections assumed that both species can fully infest all areas of predicted newly suitable habitat^4,9^. Recent trends in the global spread of these species, however, suggest that the process of expansion may be more complex and spatially structured than previously acknowledged^10^. Expansion from the native ranges of *Ae. aegypti* (from African forests) and *Ae. albopictus* (from Asia) was precipitated by a shift from zoophily to anthropophily and by adaptation to container-breeding in domestic or peridomestic environments^11,12^. While their short flight ranges limit self-powered dispersal^13^, a century of rapid human population growth and international trade has enabled their global spread. Trade in items such as tyres and potted plants have provided potential larval development habitats and have led to the intercontinental dissemination of their desiccation-resistant eggs^14–16^. Moreover, the establishment of *Ae. albopictus* in locations with cooler climates has been aided by its ecological plasticity, with eggs able to undergo diapause (dormancy) as one possible explanation for populations persisting through winters that are too cold for adult survival^17,18^.

While the various routes of intercontinental importation are well described^11,19^, the processes underlying intracontinental spread of the species remain poorly quantified, preventing an informed prediction of future distributions. Modelling of human-mediated range expansion suggests that quantitative models of human movement could, and should, be used to predict intracontinental spread^20–22^. To address this, we developed predictive models of *Ae. aegypti* and *Ae. albopictus* spread and combined these with forecasts of future climatic conditions and urban growth to predict the ranges of these medically important vectors from 2015 to 2080 (Supplementary Fig. 1).

**Fig. 1:**
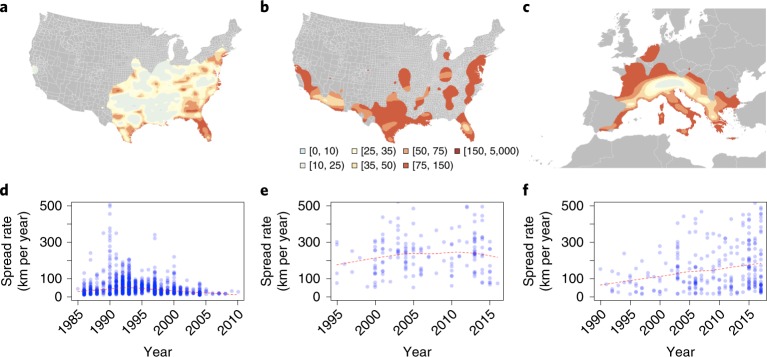
Reconstruction of *Ae. albopictus* and *Ae. aegypti* spread. **a**–**c**, Spread of *Ae. albopictus* (**a**) and *Ae.* *aegypti* (**b**) in the United States, and spread of *Ae. albopictus* in Europe (**c**). Estimates of speed of spread in km per year are based on thin spline regression on mosquito observations since their earliest detection in each continent. Red indicates fast dispersal whereas yellow and white indicate slower spread velocity measured in km per year (see legend below **b**). Areas highlighted in grey have no reported mosquito presence. **d**–**f**, Summaries of the speed of dispersal of *Ae. albopictus* (**d**) and *Ae. aegypti* (**e**) spread in the United States and of *Ae. albopictus* spread in Europe (**f**) starting from their date of first detection until 2017. The red line indicates the average velocity per year across all districts using the thin spline regression model.

We collated spatially and temporally explicit data on the distributions of *Ae. aegypti* and *Ae. albopictus* and their spread over time in the United States and of *Ae. albopictus* in Europe (Fig. 1; Supplementary Figs. 2 and 3). Extending on a previous study^4^, we first mapped contemporary habitat suitability for each species together with projected suitability in 2020, 2050 and 2080 under three different representative concentration pathways (RCPs) and 17 global climate models (GCMs), as well as under projections of urban growth. We then parameterized quantitative models of human mobility using census data on migration and commuting patterns^23,24^, and general movement patterns derived from mobile phone logs (or call detail records (CDRs))^23–25^ (Supplementary Fig. 1). The combined predictions from these different mobility models and datasets capture different aspects of human travel and trade, and their ability to spread *Aedes* eggs and juveniles at different spatial scales.

We tabulated annualized presence records that documented the first detection of each species in 1,567 different locations over 39 years in Europe (225 out of 1,588 districts between 1979 and 2017) and 32 years in the United States (1,342 out of 3,134 counties between 1985 and 2016) (Supplementary Fig. 2a–c). These data were used to parameterize statistical models of spatial spread for each species. Detection within a given area was modelled as a function of the following factors: (1) the receptivity of the area (as determined by the habitat suitability models); (2) long-distance importation pressure (from multiple human movement models); and (3) short-distance importation pressure from adjacent areas (to represent natural dispersal). Forward simulation of these fitted models of spatial spread was then used to predict the future spread or recession of each species, considering climate changes, urbanization and human-mediated importation. To account for potentially biased sampling procedures, we performed a comprehensive sensitivity analysis assuming different levels of detection for both species (Supplementary Information).

## Results

Short-range importation between adjacent districts played a greater role in the inferred spread process for *Ae. albopictus* (Fig. 1a,c,d,f) than for *Ae. aegypti* (Fig. 1b,e), which was more frequently imported over longer distances. Historically, most of the observed range expansion of *Ae. aegypti* in the United States originated from the southern states (Fig. 1b; Supplementary Fig. 2b). Using thin plate spline regression, we estimated the localized invasion velocity of *Ae. aegypti* spread in the United States to be relatively homogeneous at ~250 km per year (Fig. 1b,e). *Ae. albopictus* spread in the United States was fastest between 1990 and 1995 (Fig. 1a,d) and has since slowed to ~60 km per year. In contrast, the estimated rate of spread of *Ae. albopictus* in Europe is faster (~100 km per year), rising to ~150 km per year over the past 5 years (Fig. 1c,f; Supplementary Fig. 2c,f,i). The geographical origin of recent *Ae. albopictus* spread in Europe seems to be Italy, with the Alps serving as a dispersal barrier that lowers the rates of spread (Supplementary Fig. 2c,f). Once that barrier has been overcome, however, spread rates beyond the Alps are as high as in Italy. This may explain the increased rate of spread in recent years, which also corresponds to the detection of *Ae. albopictus* in areas north of the Alps (Supplementary Fig. 2c,f).

**Fig. 2:**
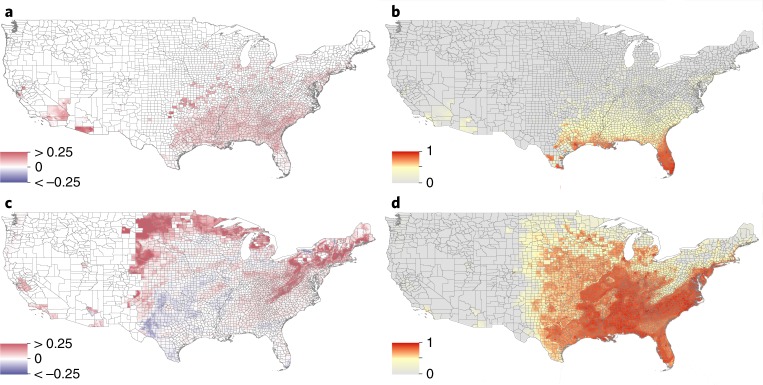
Predicted future spread of *Ae. aegypti* and *Ae. albopictus* in the United States. Spread was estimated using human-mobility metrics and ecological determinants fitted to past occurrence data. **a**, Forecasted change in the distribution of *Ae. aegypti* between 2020 and 2050 using the medium climatic scenario RCP 6.0 at the US county-level ranging from −0.25 (blue) to 0.25 (red). Red indicates expansion and dark blue contraction of the *Aedes* range distribution between 2020 and 2050. **b**, The predicted habitat suitability for the presence of *Ae. aegypti* in 2050. Pixels with no predicted suitability are in grey. **c**,**d**, The corresponding results of **a** and **b** for *Ae. albopictus*.

Using human-mobility-driven statistical models, we can predict the past spread of both mosquito species with high reliability (Supplementary Fig. 6) and accuracy (out of sample area under the receiver operating characteristic curve (AUC) value of 0.7–0.9; Supplementary Fig. 7). Compared with models that only included distance and adjacency metrics, only slight improvements are observed when including human mobility models (Supplementary Information; Supplementary Fig. 12). Furthermore, we evaluated the ability of our models to predict the range expansion in Europe using a model fitted to the US data (1,149 records) only. This test similarly documented a high degree of predictive ability (out of sample AUC value of 0.8–0.9; Supplementary Fig. 8). In addition, country borders do not seem to limit the spread of the mosquitoes (Supplementary Fig. 11), and our spread model is robust even under different assumptions in mosquito sampling strategies. However, the underlying observational data may affect our estimates of velocity of spread (Supplementary Information). In contrast, the model fitted to only European data was unable to predict the spread in the United States, presumably because of the relatively few *Ae. albopictus* records in Europe compared with the United States (192 records). Therefore, we used the model fitted to US data to project the range of both species into the future (Supplementary Information). Both *Ae. aegypti* and *Ae. albopictus* are anticipated to continue expanding beyond their current distributions (Supplementary Figs. 4 and 5). For *Ae. aegypti*, predicted future spread is mostly concentrated within its tropical range and in new temperate areas in the United States and China, reaching as far north as Chicago and Shanghai, respectively, by 2050 (Figs. 2 and 4; Supplementary Fig. 4). At the expansion front in the United States, our model predicts the spread to occur mostly through long-distance introductions in large urban areas (Fig. 2a,b; Supplementary Fig. 10). Even under the most extreme scenarios (RCP 8.5 in 2080), *Ae. aegypti* is predicted to establish in Europe in only a few isolated regions of southern Italy and Turkey (Supplementary Fig. 4). By 2080, we predict that there will be 159 countries worldwide (range, 156–162) reporting this species, of which three (range, 0–6) will be reporting it for the first time (Supplementary Table 8).

**Fig. 3:**
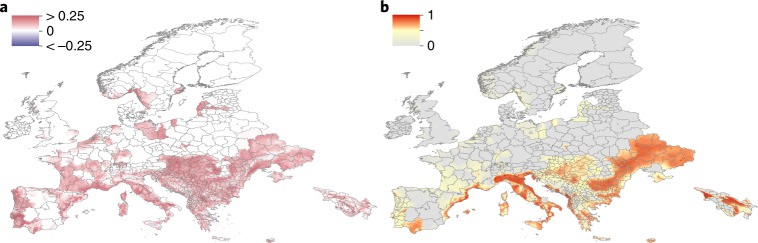
Predicted future spread of *Ae. albopictus* in Europe. **a**, The expansion (red) and contraction (blue) of *Ae. albopictus* between 2020 and 2050 under the medium climate scenario RCP 6.0, with emissions peaking in 2080. **b**, The predicted distribution of *Ae. albopictus* and predicted habitat suitability for the presence of *Ae. albopictus* in 2050. Pixels with no predicted suitability are in grey.

**Fig. 4:**
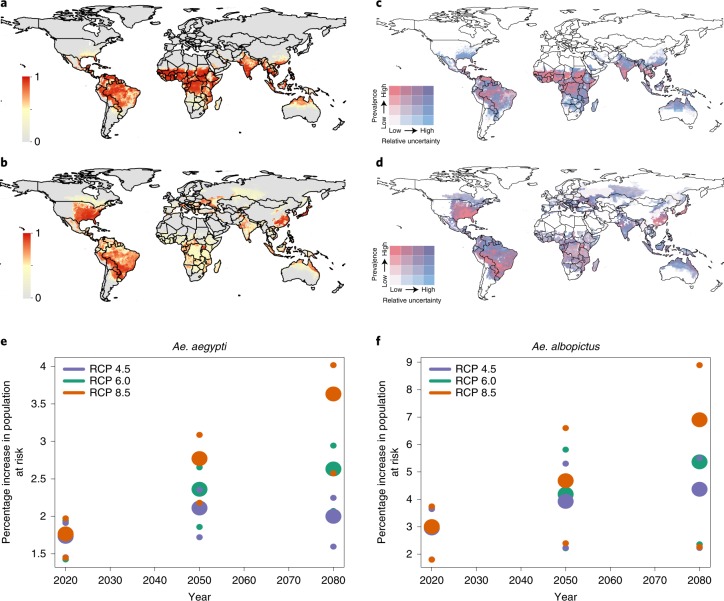
Predicted global geographical distribution of *Ae. aegypti* and *Ae. albopictus.* **a**–**d**, The distribution of *Ae. aegypti* (**a**) and *Ae. albopictus* (**b**) in 2050 under the medium climatic scenario RCP 6.0 and uncertainty for *Ae. aegypti* (**c**) and *Ae. albopictus* (**d**). Predicted habitat suitability of *Ae*. *aegypti* quantile cut-off points were 0.24, 0.66, 0.88. Relative uncertainty was computed as the ratio of the 95% uncertainty intervals and predicted *Ae. aegypti* suitability for each pixel. Cut-off points for uncertainty were 0.08, 0.18, 0.31. The lowest quantile of predicted suitability is shown in white and the highest in dark pink. The lowest quantile for uncertainty is white and the highest is blue. The colours overlap such that areas in purple have both high predicted suitability of *Ae. aegypti* and high relative uncertainty. Pixels with high predicted suitability are shown in red whereas pixels with no predicted suitability are in grey. Predicted habitat suitability of *Ae*. *albopictus* quantile cut-off points were 0.13, 0.41, 0.70. Cut-off points for uncertainty for *Ae. albopictus* were 0.16, 0.36, 0.53. **e**,**f**, The global population predicted to live in areas suitable for *Ae. aegypti* (**e**) and *Ae. albopictus* (**f**) under the conservative (RCP 4.5), medium (RCP 6.0) and worst-case scenario (RCP 8.5) using the binary cut-off values of suitability of 0.46 and 0.51 for both species, respectively.

By contrast, *Ae. albopictus* is expected to spread broadly throughout Europe, ultimately reaching wide areas of France and Germany (Fig. 3b). Areas in northern United States and highland regions of South America and East Africa are also projected to see establishment of *Ae. albopictus* over the next 30 years (Figs. 2 and 4). At the same time, some areas are predicted to become less suitable for the species, particularly locations in central southern United States (Fig. 2; Supplementary Fig. 5) and Eastern Europe (Fig. 3), where climate models indicate that aridity will increase. Due to the broader distribution of *Ae. albopictus* in northern latitudes, as in the United States, the spread pressure follows a clear front-like expansion (Fig. 2c,d). In total, 197 countries (range, 181–209) are expected to report *Ae. albopictus* by 2080, with 20 (range, 4–32) of those countries reporting its presence for the first time (Supplementary Table 8).

Spread of both species over the next 5–15 years is predicted to occur independently of extensive environmental changes, as both species continue to expand into their anthropogenic ecological niches through spatial dispersal. *Ae. albopictus* is anticipated to saturate its ecological niche between 2030 and 2050 (Fig. 4d, f), and *Ae. aegypti* by 2020 (Fig. 4a,c). Beyond these dates, the predicted expansion of these species will be driven primarily by environmental changes that create new habitats, including changes in climate, especially temperature (Supplementary Tables 1 and 2), as well as exploitation of the increased availability of large human urban environments. Thus, efforts to curb or reverse climate change are predicted to be insufficient to fully prevent the expansion of these vector species. Significantly greater expansion, however, is predicted, especially between 2050 and 2080, if emissions are not reduced (Fig. 4). At the same time, future human population growth is expected to be concentrated disproportionately within areas where *Ae. aegypti* and *Ae. albopictus* will already be established, leading to large increases in the global population at risk of diseases transmitted by these species.

Overall, our predicted expansions will see *Ae. aegypti* invading an estimated 19.96 million km^2^ by 2050 (19.91–23.45 million km^2^, depending on the climate and urbanization scenarios), placing an estimated 49.13% (48.23–58.10%) of the world’s population at risk of arbovirus transmission (Fig. 4c, f).

Few countries conduct routine, systematic surveillance for *Ae. aegypti* and *Ae. albopictus*. Consequently, our analysis relies on datasets from the United States and Europe that contain spatiotemporal biases in reporting (Supplementary Fig. 2), with an implicit assumption that the processes driving spread in these regions apply elsewhere. These regions have a comparatively high capacity to track establishment and mitigate the spread of these species, and have openly available datasets on human movement^26^. Our modelled rate of spread is therefore most likely to be biased towards an underestimate of the global rate of spread (Supplementary Information). We did not model potential changes in human mobility, which could increase the rate of spread of both species as population mobility increases. Competitive displacement may occur between these two species, but this possibility could not be included in this analysis due to a lack of available data^27,28^. However, current ecological literature and ecological theory suggests that interspecific competition occurs primarily at localized spatial scales and has not been found to influence the distributions of species at a coarser spatial resolution, such as the scale we consider here^29–31^. As both species are already established on every human-inhabited continent on the planet, we did not model spread between continents.

## Discussion

In the context of predicting mosquito-borne viral transmission, *Aedes* distribution maps have already been shown to help predict the local^32^, regional^33,34^ and international^1,2,6,7,35,36^ spread of chikungunya, dengue, yellow fever and Zika viruses. Moreover, local outbreaks of these arboviruses have typically followed within 5–15 years of infestation by *Ae. aegypti* and *Ae. albopictus*^37,38^, emphasizing the importance of vector spread importation as a key risk factor for arbovirus transmission.

There is significant uncertainty surrounding future predictions of changes in climatic conditions. We used an ensemble approach to propagate the uncertainty from climate scenarios through our predictions of both *Aedes* species (Figs. 2, 3 and 4; Supplementary Figs. 4 and 5).

Even under current climate conditions and population densities, both vector species will continue to spread globally over the coming decades, filling unoccupied suitable habitats and posing a risk to human health in the majority of locations where they survive and reproduce. Thus, efforts to prevent their global dissemination in the near future will be most effective if focused on preventing human-mediated spread and establishment. To prevent introductions, countries should strengthen entomological surveillance, particularly around high-risk introduction routes such as ports and highways, and develop rapid response protocols for vector control to prevent introduced mosquitoes from establishing permanent populations^39–43^. We expect such efforts will need to intensify over time as human populations become ever more connected and urban agglomerations grow further^9^.

Beyond 2030 and especially 2050, the distributions of both species will continue to expand, coinciding with niche expansion into climatically suitable urban areas as opposed to the exploration of the current niche. Increased urbanization worldwide has already put great strains on our ability to prevent the spread of certain disease vectors and has intensified endemic transmission of arboviruses^44^. Some areas may become less suitable for human habitation due to the effects of climate change, reducing the number of people living in areas at risk. In the longer term, reducing emissions of greenhouse gases would be desirable to limit the increase in suitable habitats for *Ae. aegypti* and *Ae. albopictus*. Every effort must be made to limit factors that contribute to the global spread of *Ae. aegypti* and *Ae. albopictus* if we are to limit the future burden of the diseases vectored by these mosquitoes.

## Methods

We used a combination of the following two approaches to estimate the predicted future distribution of *Ae. aegypti* and *Ae. albopictus*: (1) projecting the environmental suitability of both species using a set of seven environmental covariates and (2) simulating the spread within each continent using past dispersal patterns of these species, human movement data and between region adjacency matrices (Supplementary Fig. 1). Here, we describe the models and data sources for both processes.

### Data

#### Global mosquito occurrence data

We used a previously collated database of 19,930 and 22,137 geopositioned occurrence records for *Ae. aegypti* and *Ae. albopictus*, respectively^45^ (Supplementary Fig. 3). Each of these records corresponds to a unique detection of a mosquito population in a given location at a given point in time, as described in detail elsewhere^45^. We excluded records that were classified as temporary presence when such information was available.

#### Environmental and socioeconomic covariates

*Aedes* survival is influenced by a variety of climatic and environmental factors, such as long-term and interannual temperature^46,47^, water availability (described as relative humidity and precipitation) and degree of urbanization. We used projections from the RCP developed by the Intergovernmental Panel on Climate Change^48^, which represent different assumptions about emission scenarios that might result in a variety of climatic changes over the next 65 years. Here, we use RCPs 4.5, 6.0 and 8.5, which assume emission peaks around 2040, 2080 and increases throughout the twenty-first century, respectively^48^. These time points were chosen because of the following reasons: (1) 2020 represents the date when the climate-mitigating policies of the Paris Agreement within the United Nations Framework Convention on Climate Change will come into action^49^; (2) 2080 corresponds to the date of the emission peaks modelled according to the RCP 6.0 scenario; and (3) 2050 represents the midpoint between these dates. We use an ensemble of 17 GCMs and pattern scaling to produce monthly mean values of maximum and minimum temperature and monthly totals of rainfall as used in MarkSim. Humidity data were calculated from temperature estimates (for details, see the “Future projections” section). To complement the changes in temperature, relative humidity and precipitation, we modelled a continued process of global urbanization until 2080 using a probabilistic machine learning algorithm based on a previously described method^50^. Here, we used urban growth rates projected by the United Nations as a predictor variable^51^ as well as a range of other critical covariates, as described previously^50^.

#### Mosquito spatial spread data

A unique set of time-series occurrence records for both species were abstracted from previous studies^4,45^ and updated with records obtained from another published study^52^. Records were available for *Ae. aegypti* in the United States from 1995 to 2016, with US county-specific information regarding whether the species was present or absent. For *Ae. albopictus*, information was available from the United States (1985–2013) and from Europe (1979−2017) (Fig. 1; Supplementary Fig. 2). We considered these time periods because they show consistent expansion of the species distribution, as described previously^52^.

For the United States, counties were identified as reporting the presence of either species in a given year if at least one specimen of any life stage of the mosquito was collected, using any collection method^52^. Sampling efforts, techniques and temporal resolution were heterogeneous across counties and states in the United States. Therefore, the baseline presence datasets may classify some areas as absent where either of the two *Aedes* species considered may be present.

For Europe, Administrative/Statistical units (NUTS3) were identified as reporting establishment of either species in a given year if immature stages and overwintering were observed, using any collection method. Sampling efforts, techniques and temporal resolution were heterogeneous across countries, and either species may have been absent before investigations were triggered by citizen complaint. Therefore, dates correspond to published reports or expert-shared data (VBORNET and VectorNet), and a species could have established earlier in some locations where regular surveillance had not been implemented. Because we were not able to quantify the sampling biases, we instead employed a sensitivity analysis to account for potential under- or over-reporting (see the “Mosquito spread modelling” section).

#### Human mobility datasets

Overland human movements are known to drive the importation of both species^40,41,43^. Therefore, we used human movement data to infer the connectivity between regions as a proxy for importation risk of *Ae. aegypti* and *Ae. albopictus*.

##### US commuting data

For the United States, where both species have been spreading successfully, we obtained data on workforce commuting flows from county to county between 2009 and 2013 conducted by the American Community Survey. Data are freely available at http://www.census.gov/hhes/commuting/. Here, commuting was defined as a worker’s travel between home and workplace, where the latter refers to the geographical location of the worker’s job. Daytime population refers to the estimated number of people who are residing and working in an area during ‘daytime working hours’. The data represent 3,134 counties, including 50 states and the District of Columbia but excluding Puerto Rico. The generalizability of this dataset has been demonstrated in studies that have successfully approximated human movements derived from mobile phone data and predicted the spread of infectious diseases^24^. As described in detail below in the “Human mobility modelling” section, we considered gravity and radiation movement models as well as nearest neighbour-type movements for human movement. We used the fitted models from the United States to extrapolate to all other regions in the Americas using the movement package in R^53^.

##### European mobile phone data

For Europe, we obtained mobile phone data (or CDRs) from the following three countries where *Ae. albopictus* is present or has recently been detected: France^45^, Portugal^54^ and Spain^45^. CDR data contain the time at which a call was made or a text message was sent, the duration of the call, and the code of the mobile phone in which communication started. The mobile phone corresponds to an area covered by a specific mobile phone tower that serves a particular area. This means that the spatial resolution is restricted to the tower area, and the specific location of each individual in the dataset cannot be ascertained. As our analysis was performed at the district level, all users’ activity profiles were aggregated up to the district level, which is generally larger than mobile phone tower areas. We thereby obtained a connectivity matrix that shows the connections made between each district *i* to each district *j* within each respective country.

For Portugal, data were available from over 1 million mobile phone users between April 2006 and March 2007 (12 months). In Spain, CDRs were extracted from 1,034,430 users over 3 months between November 2007 and January 2008. In France, we had the largest sample of 5,695,974 users, collected between September 2007 and mid-October 2007, covering the entire country. Other aspects of the collection and processing methods have been described in detail elsewhere^23^. We used the fitted models from Europe to extrapolate to all other regions in Europe, using the movement package in R^53^.

##### Human movement data for Asia

Mobility matrices for Asia were inferred from data from Chinese users of Baidu, the largest location-based service (LBS) in China. Baidu offers a large variety of apps and software for mobile devices and personal computers, mostly for online searching. We extracted global positioning system (GPS) data from 23 April 2013 to 30 April 2014 (about 400 million users in China). The raw data were collected at the county level (*n* = 2,959) and aggregated to the prefecture level (345 prefectures). We then estimated daily flows of people between each pair of counties and aggregated this information per year. Movement is recorded in the Baidu data such that on each day, if a user was observed at locations A→B→C, then A→B and A→C are counted, which may produce biased population flow estimates. To explore potential bias in the data, we compared the data derived from Baidu to a complete dataset of taxi-based GPS locations in the capital city of Hunan province, covering a 1-week period (full details below). The correlation of origin-to-destination flows in the city between the Baidu data and the complete taxi GPS data was very high (*R*^2^ = 0.99).

##### Baidu data validation

To verify the validity of the Baidu LBS data, we obtained a complete dataset of GPS locations for all taxis in Changsha city (capital of Hunan province, with a population of 7 million) in 2014. For regulatory reasons, the location of each taxi is recorded using a GPS device in each taxi. The location is updated every 30 s. There were approximately 7,000 taxis in Changsha, resulting in 20.16 million records (7,000 × 24 × 60 × 2) on a daily basis. The status of the taxi was also recorded, such as the locations where passengers got on and off. These data were then used to extract the movements between the following five districts in the main area of Changsha: Kaifu, Furong, Yuhua, Tianxin and Yuelu. For the purpose of comparison, 1 week of data (4 April to 17 April 2016) were extracted and analysed. The movements were normalized and then compared with the same week in 2014 from the Baidu LBS data. The correlation between the mobility estimates extracted from the Baidu LBS data and from the taxis’ GPS data for Changsha city is presented in Supplementary Fig. 9. There was a high level of similarity between the two datasets, with a correlation coefficient of 0.99 (*P* = 0.001). We subsequently used the fitted models from China to extrapolate to other regions in Asia and Oceania, again using the movement package in R^53^.

##### Human movement data for Africa

To calibrate the gravity and radiation models for Africa, we used aggregated and de-identified mobile phone-derived mobility estimates at the constituency level from Namibia between 1 October 2010 and 30 September 2011. These data represent the proportion of time that unique subscriber identity module (SIM) cards in each constituency spend in all other constituencies, as previously described in detail^55^. We used this dataset from Namibia because it was openly available and because it offered the best spatial and temporal resolution compared to census-derived data. We then used the fitted models to extrapolate to all other regions in Africa using the movement package in R^53^. Systematic surveys of cross-border human movements were not available at the time of the study and for the study regions.

It is possible that there are significant differences between regions in terms of mobility, but unfortunately no sufficiently widespread and well-resolved data source was available to test this. Our model captured the spread process of *Aedes* mosquitoes using a variety of human movement data, including both CDR data and commuting data. To assess the generalizability of our results, we applied the model fitted to commuting data in the United States to the range expansion process observed in Europe. The predictive ability of this cross-continental validation indicates that the mobility data used are sufficiently robust in the context of this study (Supplementary Fig. 8). However, we note that there may be several limitations to using commuting data to infer vector introductions as they overly emphasize work-related movements. To test whether our model would perform well even in the absence of human movement data, we performed a cross validation that used only distance and adjacency matrices; this process only marginally reduced predictability (Supplementary Fig. 12). Despite this, such data have indeed been used in the United States to successfully predict the long-distance spread of infectious diseases. We are therefore confident that such data can be applied to predict both short- and long-distance spread in the United States^56^. Similarly, CDR data have been used to describe the spread of pathogens such as influenza in Europe^23^. As new data become available, our model is flexible enough to incorporate them, and estimates of the predicted range expansion of *Ae. aegypti* and *Ae. albopictus* can be updated. There was also no suitable data available on cross-border movements that could improve estimates of between-country spread (see the “Mosquito spread modelling” section for a sensitivity analysis).

### Model fitting to data

#### Description of speed of dispersal

To understand the past range expansion of both species and to provide basic summary statistics of the speed of dispersal over time in areas where sufficient observations were available, we used previously described methods of spread rate measurements^57^. For each species and study area, the centroids of the spatial units where the species were observed were re-projected in a metric system (EPSG Geodetic Parameter Dataset 102003 in the United States, and EPSG 3035 in Europe), and the first date of detection in each centroid was interpolated on a 10-km resolution grid using thin plate spline regression. The local slope of the surface was measured using a 3 × 3 moving windows filter, and the resulting friction surface (time/distance) was smoothed using an average 11 × 11 cell filter to prevent local null frictions values. The local spread rate was then obtained by taking the inverse of the friction value. This measure was computed within a mask, which was obtained by kernel density smoothing of the centroids of spatial units where the species were observed. We used a previously described method^58^ to determine the optimal bandwidth for the US and European invasions. To obtain a similar bandwidth for all masks, we used the maximum of the three estimated optimal bandwidths, which was found to be 73.2 km. A density threshold of 2.9 points per 10,000 km^2^ was chosen to delineate the mask, which was the maximum threshold value that allowed the inclusions of all observation points in the mask in both the United States and Europe.

#### Mosquito environmental niche modelling

To predict the likely future distributions of both species independently (in years 2020, 2050 and 2080), we first fitted species distribution models to data from the present day. This approach built on previous work^4^ using the boosted regression tree (BRT) models fit to mosquito occurrence data (see “Global mosquito occurrence data” section). BRTs combine strengths from regression trees and machine learning (gradient boosting) and are able to accommodate nonlinear relationships to identify the environmental niche in which the environment is suitable for the species in question. After an initial regression tree is fitted, it is iteratively improved in a forward stepwise manner (boosting) by minimizing the variation in the response variable not explained by the model at each iteration. This approach has been shown to simultaneously fit complex nonlinear response functions efficiently while guarding against over-fitting.

We first developed a baseline scenario for the year 2015 using the global dataset of *Ae. aegypti* and *Ae. albopictus* occurrence (see “Global mosquito occurrence data” section)^45,59^ and a set of environmental and socioeconomic predictors (see “Environmental and socioeconomic covariates” section). In a BRT modelling framework, pseudo-absences need to be generated to allow for discrimination between areas where the mosquitoes can persist and to identify biases in reporting^60^. We used a previously described approach^4^ using background points from the Global Biodiversity Information Facility and the inverse of an *Aedes* temperature suitability mask^47^ with an equal ratio between presence and absence points and no threshold applied. From that, we constructed 100 submodels to derive the mean prediction map and model-fitting uncertainty using the SEEG-SDM package in R^61,62^.

#### Human mobility modelling

Given the heterogeneous abundance of both species^63^ as well as the low probability of their surviving slower and longer transits, the chance of a species being introduced following any single translocation event is low. Hence, we used relatively long time steps (yearly) and generalized human movement models fitted to a variety of data sources to understand the spatial spread patterns of *Ae. aegypti* and *Ae. albopictus*.

We incorporated three distinct human movement models that act at different scales, since we were uncertain a priori which type of human movement would be most associated with mosquito spread. We considered the following models: (1) a gravity model; (2) a radiation model; (3) an adjacency network model; and (4) untransformed great-circle distance. Each of these models has been shown to be useful depending on the local context to infer regular daily commuting patterns, longer-term movements and as general descriptions of human mobility^24,64,65^. First, the gravity model assumes that fluxes between two areas *i* and *j* are $T_{\mathrm{i,j}} = k\frac{{N_i^\alpha N_j^\beta }}{{d_{\mathrm{i,j}}^\gamma }}$, where *N* represents the human population size and *d* is the great circle distance between two locations, and *k*, *α*, *β* and *γ* are parameters to be fit^66,67^. The gravity model emphasizes the attractive power of large population centres. Second, the radiation model assumes fluxes to be $T_{\mathrm{i,j}} = T_i\frac{{N_iN_j}}{{\left( {N_i + s_{\mathrm{i,j}}} \right)\left( {N_i + N_j + s_{\mathrm{i,j}}} \right)}}$, where *T*_*i*_ is the number of individuals leaving area *i* and *s*_i,j_ is the total population in the circle centred at *i* with radius *d*_i,j_ excluding the population of the two areas *i* and *j*. The radiation model considers not only distance and population sizes at the origin and destination but also the cumulative population at a lesser distance from the origin than the destination^24^. Consequently, this model considers not only the origin and destination but also the landscape of ‘intervening opportunities’ between them. Third, adjacency networks encode the number of district borders an individual would need to cross to move from one district to another. Thus, this metric reflects the neighbourhood effect. Finally, we computed the great-circle distance between each pair of locations and used that as a metric of mobility in and of itself^32,68^.

For each second administrative unit (county/municipality) in the world, we determined the total human population size using gridded population estimates and calculated the great-circle distance between the centroids of each pair of districts within each continent^69^. Gravity and radiation model parameters were fitted using maximum likelihood methods to the empirical data described above using the movement R package^53^. National adjacency networks were computed using administrative boundary data from the GADM dataset (http://www.gadm.org). To account for neighbourhood effects of spread and for the potential importance of within-country and between-country movements, we constructed adjacency matrices that were disaggregated into three binary connectivity matrices with connectivity degrees of one (that is, districts share a border), two (that is, districts share a common neighbour) and three (that is, more than two degrees away).

#### Mosquito spread modelling

Let *x*_*i*_(*t*) be the *Aedes* population status of district *i* at time *t* (that is, a binary variable takes the value 1 if there were *Aedes* mosquitoes that time, and 0 otherwise). Given the nature of the dataset collected, we assumed that all data points represented detection of established populations and thus assumed continuous presence of the species for the first and last reported occurrences. We used a standard logistic model to characterize the probability that some district *j* will become occupied at time *t*:

$$\log {\mathrm{it}}\left( {P\left( {x_j\left( t \right) = 1\left| {x_j} \right.\left( {t - 1} \right) = 0} \right)} \right) = \beta _0 + \mathop {\sum }\limits_{k = 1}^n \beta _kY_{\mathrm{j,t}}^{(k)}$$

where $Y_{\mathrm{j,t}}^{(k)}$ corresponds to the value of explanatory variable *k* in district *j* at time *t*. Explanatory variables included in this analysis were the predicted vector habitat suitability (that is, suitability for establishment of an introduced vector; see “Description of speed of dispersal” section) and connectivity between infested and non-infested districts (that is, probability of introduction of a vector). Separate metrics of connectivity were defined for each human movement model (see “Mosquito environmental niche modelling” section). From each human movement model, a connectivity matrix $A_{\mathrm{ij}}^{(k)}$ was calculated for each location *i* and *j*. A corresponding covariate for the occupation model was then computed to represent the global force of importation, exerted from all other infested districts to *j*: $Y_{\mathrm{j,t}}^{(k)} = \mathop {\sum }\limits_i A_{\mathrm{ij}}^{\left( k \right)}x_i\left( {t - 1} \right)$.

These models were re-fit in each successive year separately for the North American and European datasets, and for each vector species, using all available data up to that year. Model selection was done through backward selection using Akaike information criterion^70^. The fitted model was then evaluated prospectively over the next year by comparing predicted presence or absence with observations, thereby allowing us to evaluate and validate model performance over time. For model evaluation, we considered all locations (that is, 3,134 counties in the United States, 1,587 NUTS in Europe). This model evaluation was used to identify the best explanatory variables to include in the *Aedes* spread model. Model evaluation was performed using receiver operating characteristic (ROC) curves (Supplementary Fig. 7), and model accuracy was characterized by comparing the predicted probabilities of first detection compared with the response (Supplementary Fig. 6). We calculated the probability of first detection *p*_w_ predicted by the model for each district-year that had not yet reported mosquitoes. We then partitioned district-years into eight groups, with predicted probabilities in the range of 0–1%, 1–5%, 5–10%, 10–15%, 15–20%, 20–25%, 25–35% and 35–100%. For each group, we calculated the mean predicted probability and compared it with the proportion of district-years in the group in which range expansion was observed. Our model assumes that each mosquito species will persist in an area once detected, while there are some examples of incursions apparently having been successfully eradicated or died out. It is possible that this assumption could result in inflated predictions of the rate of spread due to an overestimated number of source populations for each potential invasion event. However, it should be noted that this overestimate of the number of source populations would also be present in the training data, and would be at least partially absorbed into estimates of the probabilities of importation. Insufficient data were available to test or account for this potential bias, but based on additional experiments, we do not anticipate our estimates to greatly overpredict *Aedes* presence (see “Sensitivity analyses and sampling bias” section).

#### Cross-validation

To test whether the spread between countries is different from the spread within countries, we used the multi-country dataset from *Ae. albopictus* in Europe and varied the relative frequency of within- and between-country mobility by decreasing movement between countries by 20%, 50% and 70%. The results were then compared with a baseline, in which predicted within-country movement is the same as between-country movement (Supplementary Fig. 11). We also performed sensitivity analyses to evaluate how a model including human movements compares to single variable models that have objective measurements such as great-circle distance and adjacency. A model that includes human movements only slightly increased the predictive performance (Supplementary Fig. 12).

#### Sensitivity analyses and sampling bias

Surveillance efforts to detect *Ae. aegypti* and *Ae. albopictus* may vary in time and space due to gradual progressive improvements as a result of technology trapping technology, general expertise or in response to specific events. The following three types of changes in surveillance could bias the estimates of our spread model: (1) spatial expansion of surveillance system coverage to new areas; (2) intensification of sampling effort within areas where the surveillance system already operates; and (3) changes in sampling methods within areas where the surveillance system already operates that make it more or less likely to detect either *Ae. aegypti* or *Ae. albopictus*. To address each of these, we completed sensitivity analyses to understand how possible changes in surveillance may affect the inference about spread in the future.

Expansions of the surveillance system can be definitively distinguished from true known expansions of the vectors by comparing the state transitions of areas in longitudinal datasets, such as our *Ae. albopictus* dataset in Europe between the years of 2013 and 2017. Areas that first report absence of the species (often for multiple years) and later report presence are as close to a clear example of introduction as possible and give a reasonable estimate of the arrival date. Conversely, if an area’s first report is presence of the species, the species’ arrival date may have been estimated later than it truly occurred.

First, the existence of such longitudinal records in the *Ae. albopictus* database in Europe provides strong evidence to indicate that the distribution of the species is expanding. However, to test whether expanding surveillance efforts is a contributing factor to the observed rate of spread, we compared our original model fit to the full *Ae. albopictus* in Europe dataset, as used in our main analysis (model 1), with a model fit only to the data points that have strong evidence for a specific introduction date (that is, reported absence before presence; model 2). We tabulated data from *Ae. albopictus* in Europe where information was available regarding whether there was ongoing surveillance before the reporting of the species (transition from absence to presence). Such data were available for 179 out of 600 observations between 2013 and 2018, a time period where 400 new regions reported the presence of the species, thus making our subsample about 50% of all new invasions. This dataset was available at higher spatial resolution than for the full *Ae. albopictus* dataset for Europe. A total of 75% of these records were from locations of most recent spread in France and Germany. Finally, as model 2 was fit to data from a narrower date range, we also considered a third model (model 3), which was fit to both occurrence and longitudinal data but only from the more recent date range (Supplementary Table 3). If expansion of surveillance efforts is a contributing factor to the observed rate of spread in the data, then we would expect model 2 to predict a significantly lower rate of spread than models 1 or 3 (our null hypothesis).

Each of these models were fit to the above datasets, then used to simulate *Ae. albopictus* spread from a common baseline (based on occurrence and longitudinal data at the end of 2012) for 6 years between 2013 and 2018 as described previously. The predicted total number of new districts infested during this period was calculated and is shown in Supplementary Table 4. Note that comparison of goodness of fit metrics for these models was not possible since the models were fit to different datasets.

Contrary to the expectation that more precise dates of invasion would lead to conclusions of slower rates of spread, this sensitivity exercise found that restricting the model to just areas where the date of introduction is known significantly increases the predicted rate of spread. Thus, this exercise rejects our above null hypothesis. This effect was also independent of the time period of the fitting data (similar results were obtained for models 1 and 3). These results suggest that it is more likely that true spread of *Ae. albopictus* is outpacing expansion of mosquito surveillance, and if longitudinal surveillance was in place everywhere, the observed rates of spread would be greater.

We therefore believe that the currently implemented model is a conservative estimate of the spread of these species and it is not highly affected by changes in spatial coverage of surveillance systems. Moreover, it provides the most robust estimates of spread over these time periods given the available data. Given the limited number of years of data available to fit model 2, we believe that model 1 provides the most reliable estimates of future spread.

Intensification in sampling effort and technological advancements in collection methods may affect the probability of detection of a species earlier in their invasion process compared with today. Here, we tested both hypotheses by including different terms in our spread model regression and compared such models to the null of no changes in surveillance intensity over time (as currently implemented in our main analysis). To represent increases or decreases in surveillance over time, we included the spline-smoothed year of detection as a variable in the regression analysis. To represent step changes in surveillance efforts in response to specific events, we included a factor variable; either before the 2003 peak in West Nile virus cases in the United States or after 2003 (only for models in the United States). Internal cross-validation was then used to compare the predictive performance of these three models with evaluation on 3-year-lookahead holdout sets, subject to a minimum of 10 consecutive years of data to fit the models. Model predictive performance was then compared using deviance from observed values in the holdout set.

This process showed that for all species in all continents, the inclusion of a temporal (Year) term reduced predictive accuracy (increased deviance). This was the case for both gradual change over time (s(Year)) and for breakpoint changes in response to specific events (Year > 2003). As a result, we conclude that there is no evidence for temporal changes in sampling effort in any of the datasets concerned; therefore, we did not include such terms in our final predictions (Supplementary Table 5).

Finally, there is a possibility that changes in general vector surveillance strategies could have led to changes that affected the probability of detection of one species more than the other. Such differential biases could undermine our interspecies spread rate comparison. One key period of concern is around the 2003 West Nile virus outbreak in the United States, where vector surveillance may have prioritized trapping in more rural environments to optimize detection of various *Culex* species. Such a focus on rural environments may have led to relative increases in sampling intensity of *Ae. albopictus* and relative reductions in sampling intensities for *Ae. aegypti*.

To test this hypothesis, we followed a similar approach to the above analysis, whereby covariates for ‘before’ and ‘after’ the 2003 West Nile virus outbreak were included in the US spread model for each species. If the above hypothesis is true, such terms should have larger after values than before values in the *Ae. albopictus* model and vice versa in the *Ae. aegypti* model. Moreover, the terms should improve model prediction accuracy.

The best fits from the *Ae. aegypti* and *Ae. albopictus* spread models in the United States showed that detection of *Ae. aegypti* marginally increased relative to *Ae. albopictus* (positive model coefficients for post-2003 term in *Ae. aegypti*, negative in *Ae. albopictus*) (Supplementary Table 6). However, as previously stated, inclusion of such changes in surveillance quality over time reduced the model predictive performance (increase in deviance for both species) and therefore may not provide a better time period to mirror the spread of the species in the United States.

#### Classifying the ranges of each mosquito species and incorporating uncertainty

Current reported distributions of *Ae. aegypti* and *Ae. albopictus* are unlikely to be fully representative of their actual distributions because of logistical and financial constraints on vector surveillance^39^. Therefore, we used the following method to estimate the current-day global distribution (realized niche) of each mosquito species by comparing environmental suitability maps with occurrence data. We extracted the predicted environmental suitability value at each of the locations where the mosquito species has been reported, and the value of environmental suitability that encompassed 90% of these reported locations was chosen as the range threshold. Every value above or equal to this threshold was defined as within the range of the mosquito species (Supplementary Fig. 13). This approach assumes that the 10% of occurrences outside the predicted range represent temporary introductions that do not persist longer than 1 year and are not representative of the long-term distribution of the species. As there is uncertainty regarding what proportion of the data are representative of these transient identifications (given that the majority of the data are cross-sectional not longitudinal), we undertook a sensitivity analysis that varied this threshold from 85% to 95%, thereby creating 96 different possible range maps that represent different realizations of the current distribution of each species. In doing so, we captured locations that have the conditions for mosquito presence and where there is potential for onward spread. We did not include international shipping as a contributor to infrequent long-distance importation events between continents since both species are already well established on each continent; therefore, new occurrences are more likely to be driven by intracontinental importation pressure.

### Future projections

#### Projecting environmental and socioeconomic covariates

We used 17 GCMs to estimate 30 arcsec images for monthly mean climate data. Supplementary Table 7 provides the designation, origin, references and number of replicate runs for each model. The procedures are described in detail in MarkSim documentation^65^. For each GCM, the baseline monthly climate was derived from the historical runs for temperatures and rainfall, the monthly means were calculated for each GCM for the years 2000 to 2095, and the difference ‘delta’ for each month was calculated by subtracting the specific GCM baseline. The delta values were interpolated from the native GCM pixel (Supplementary Table 7) to a one degree by one degree pixel for the globe. The data were pattern scaled to WorldClim 1.03^64^ for each one degree pixel, RCP and month. For each variant, a fourth-order polynomial regression was fitted over the 96 years of data and through the origin at 1985 (1985 being the mean midpoint of the data used in the WorldClim construction) to calculate one output per model per year per scenario.

Humidity data were estimated directly at the 30 arcsec level from dewpoint calculated using a previously described tabular method^71^ and the mean temperature. To fully propagate the variation between the climate models through our predictions, we used the outputs of 17 GCM for all 3 years and 3 scenarios.

Global temperature estimates were converted into temperature suitability for mosquito population persistence (separate metrics for each vector species), hereafter referred to as temperature suitability, using previously described temperature-based mathematical models^44,46,47^. These show the effects of diurnal and seasonal changes in temperatures on the generation time of the mosquito and its resultant effects on the persistence of a population.

As a highly anthropophilic mosquito species, the future distribution of the *Aedes* is likely to depend critically on both environmental and human socioeconomic factors that modify the availability of its habitat^8^. To incorporate these features, we also modelled the continued process of global urbanization until 2080 using a probabilistic machine learning algorithm based on a previous study^50^. Here, we used urban growth rates predicted by the United Nations as a predictor variable^51^ as well as a range of other covariates as previously described^50^.

#### Projecting future niches of *Ae. aegypti* and *Ae. albopictus*

Although niche shifts might occur over long time-periods, the future effects remain unclear for *Ae. aegypti* and *Ae. albopictus* since their expansion from their native range^72^. Therefore, we assume niche conservatism, implying that the mosquitoes tend to establish and survive under similar environmental conditions in native and invaded ranges in the future^4,73,74^.

Our final aim was to produce 18 maps predicting *Ae*. *aegypti* and *Ae. albopictus* habitat suitability in the years 2020, 2050 and 2080 under three different emissions scenarios (RCPs). Each of these 18 maps were composed of 100 ensemble predictions that randomly sampled (with replacement) the following aspects of the analysis:

1. The fitted *Aedes* BRT model (from a choice of 100 BRT models fitted to 2015 data).
2. The predicted temperature suitability for *Aedes* survival (from a choice of 17 GCMs).
3. The predicted minimum precipitation (from a choice of 17 GCMs).
4. The predicted relative humidity (from a choice of 17 GCMs).
5. The predicted maximum precipitation (from a choice of 17 GCMs).
6. The predicted geographical expansion via land from the spread models (see “Projecting mosquito spread” section).

This approach sought to fully propagate the uncertainty in the climate, *Aedes* temperature suitability and *Aedes* models through to the final prediction. These 100 predictions were then summarized by mean and 95% credible intervals to give the final prediction for each year RCP combination. Uncertainties are shown in all maps along the *x* axes.

Our baseline map modelling is different from previously published maps in that it uses only projectable environmental and sociodemographic variables and does not use the enhanced vegetation index, as this index is a direct empirical measure of the Earth’s current greenness^4^. To minimize potential reduction in the predictive ability of the model by omitting this covariate, we included precipitation and relative humidity as predictors for suitability for green vegetation growth in both the present day and future models.

#### Projecting mosquito spread

To derive yearly model-based estimates of the possible expansion of both species by 2080, we forward-simulated the geographical spread model based on the equation in the “Mosquito spread modelling” section. To account for the spatiotemporal dependence in first detection probabilities (each district’s probability is a function of every other district that was infested the year before), we ran 1,000 simulations forward in time. Within each simulation, we estimated the probability of infestation to each district that had yet to detect the species. We then drew a Bernoulli random variable with that probability of 1 (that is, invasion) and imputed those results for each potential detection. Using these imputed invasions as well as all districts that had previously been infested, we repeated the estimation of range expansion for the next year. This process was repeated up to the desired forecast horizon. This represents a single simulation. It is important to note that we did not allow for the situation where an already infested district will ‘lose’ its infection status (that is, if *x*_*i*_(*t* − 1) = 1 for district *i*, we force *x*_*i*_(*t*) = 1). We then combined the results of the 1,000 simulations to identify which districts were most likely to have a positive species presence at any point.

#### Calculating population at risk and area expansion

To classify areas as at risk or not at risk of *Ae. aegypti* and *Ae. albopictus* expansion, a threshold was defined for the continuous *Aedes* suitability maps by the value that maximized sensitivity and specificity when classifying the occurrence and background data using the 2015 map. This value was found to be 0.47 and 0.51 for *Ae. aegypti* and *Ae. albopictus*, respectively. Any pixel with a predicted suitability value above that was considered at risk, and the same threshold was applied to each time point and scenario to calculate the population and area at risk in each global region. The final maps for 2020, 2050 and 2080 were then overlaid with contemporary estimates of human populations at 5-km resolution and extracted the relevant population at risk was estimated using the raster package in R. We paired the climatic scenarios based on shared socioeconomic pathways (SSPs) that were defined previously^75^. They represent reference pathways that describe plausible alternative trends in the evolution of society and ecosystems over a century, in the absence of climate change or climate policies. SSPs are predicated on possible outcomes that would make it more or less difficult to respond to climate change challenges. Each SSP consists of quantified population and gross domestic product (GDP) trajectories, serving as the starting points for various organizations to model these factors and to provide projections for demographic and economic development variables. The Integrated Assessment Modelling Consortium made available certain peer-reviewed projections via the International Institute for Applied Systems Analysis (http://www.iiasa.ac.at), whereby the SSP storylines were converted into population and GDP projections for 195 countries^76^ for every decade between the years 2010 and 2100.

### Reporting Summary

Further information on research design is available in the Nature Research Reporting Summary linked to this article.

## Supplementary information

Supplementary Information Supplementary Notes, Supplementary Figures 1–13, Supplementary Tables 1–9 and Supplementary References.

Reporting Summary

## Data Availability

Data are available from https://datadryad.org/resource/doi:10.5061/dryad.47v3c
