# Publisher Correction: Past and future spread of the arbovirus vectors *Aedes aegypti* and *Aedes albopictus*

**DOI:** 10.1038/s41564-019-0429-2

**Published:** 2019-03-21

**Authors:** Moritz U. G. Kraemer, Robert C. Reiner, Oliver J. Brady, Jane P. Messina, Marius Gilbert, David M. Pigott, Dingdong Yi, Kimberly Johnson, Lucas Earl, Laurie B. Marczak, Shreya Shirude, Nicole Davis Weaver, Donal Bisanzio, T. Alex Perkins, Shengjie Lai, Xin Lu, Peter Jones, Giovanini E. Coelho, Roberta G. Carvalho, Wim Van Bortel, Cedric Marsboom, Guy Hendrickx, Francis Schaffner, Chester G. Moore, Heinrich H. Nax, Linus Bengtsson, Erik Wetter, Andrew J. Tatem, John S. Brownstein, David L. Smith, Louis Lambrechts, Simon Cauchemez, Catherine Linard, Nuno R. Faria, Oliver G. Pybus, Thomas W. Scott, Qiyong Liu, Hongjie Yu, G. R. William Wint, Simon I. Hay, Nick Golding

**Affiliations:** 1 Department of Zoology, University of Oxford, Oxford, UK; 2 Harvard Medical School, Harvard University, Boston, MA USA; 3 Boston Children’s Hospital, Boston, MA USA; 4 Institute for Health Metrics and Evaluation, University of Washington, Seattle, WA USA; 5 Centre for Mathematical Modelling of Infectious Diseases, London School of Hygiene and Tropical Medicine, London, UK; 6 Department of Infectious Disease Epidemiology, London School of Hygiene and Tropical Medicine, London, UK; 7 School of Geography and the Environment, University of Oxford, Oxford, UK; 8 Oxford School of Global and Area Studies, University of Oxford, Oxford, UK; 9 Spatial Epidemiology Lab (SpELL), Universite Libre de Bruxelles, Brussels, Belgium; 10 Fonds National de la Recherche Scientifique, Brussels, Belgium; 11 Department of Statistics, Harvard University, Cambridge, MA USA; 12 RTI International, Washington, DC USA; 13 Epidemiology and Public Health Division, School of Medicine, University of Nottingham, Nottingham, UK; 14 Department of Biological Sciences and Eck Institute for Global Health, University of Notre Dame, Notre Dame, IN USA; 15 School of Health, Fudan University, Key Laboratory of Public Health Safety, Ministry of Education, Shanghai, China; 16 Department of Geography and Environment, University of Southampton, Southampton, UK; 17 Flowminder Foundation, Stockholm, Sweden; 18 School of Business, Central South University, Changsha, China; 19 College of Systems Engineering, National University of Defense Technology, Changsha, China; 20 School of Business Administration, Southwestern University of Finance and Economics, Chengdu, China; 21 Waen Associates Ltd, Y Waen, Islaw’r Dref, Dolgellau, Gwynedd UK; 22 Pan American Health Organization (PAHO), Washington, DC USA; 23 National Dengue Control Program, Ministry of Health, Brasilia, Brazil; 24 European Centre for Disease Prevention and Control, Stockholm, Sweden; 25 Institute of Tropical Medicine, Antwerp, Belgium; 26 Avia-GIS, Zoersel, Belgium; 27 Francis Schaffner Consultancy, Riehen, Switzerland; 28 Department of Microbiology, Immunology, and Pathology, Colorado State University, Fort Collins, CO USA; 29 Computational Social Science, ETH Zurich, Zurich, Switzerland; 30 Department of Public Health Sciences, Karolinska Institutet, Stockholm, Sweden; 31 Stockholm School of Economics, Stockholm, Sweden; 32 Insect–Virus Interactions Unit, Institut Pasteur, CNRS, UMR2000 Paris, France; 33 Mathematical Modelling of Infectious Diseases Unit, Institut Pasteur, CNRS, UMR2000 Paris, France; 34 Department of Geography, Universite de Namur, Namur, Belgium; 35 Department of Entomology and Nematology, University of California, Davis, Davis, CA USA; 36 State Key Laboratory of Infectious Disease Prevention and Control, Collaborative Innovation Center for Diagnosis and Treatment of Infectious Diseases, National Institute for Communicable Disease Control and Prevention, Chinese Center for Disease Control and Prevention, Changping, Beijing, China; 37 Shandong University Climate Change and Health Center, School of Public Health, Shandong University, Jinan, Shandong China; 38 WHO Collaborating Centre for Vector Surveillance and Management, Beijing, China; 39 Chongqing Centre for Disease Control and Prevention, Chongqing, China; 40 Environmental Research Group Oxford (ERGO), Department of Zoology, Oxford University, Oxford, UK; 41 School of BioSciences, University of Melbourne, Parkville, Victoria, Australia

**Keywords:** Microbiology, Infectious diseases

Correction to: *Nature Microbiology* https://doi.org/10.1038/s41564-019-0376-y, published online 4 March 2019.

In the version of this Article originally published, the affiliation for author Catherine Linard was incorrectly stated as ‘^6^Department of Infectious Disease Epidemiology, London School of Hygiene and Tropical Medicine, London, UK’. The correct affiliation is ‘^9^Spatial Epidemiology Lab (SpELL), Universite Libre de Bruxelles, Brussels, Belgium’. The affiliation for author Hongjie Yu was also incorrectly stated as ‘^11^Department of Statistics, Harvard University, Cambridge, MA, USA’. The correct affiliation is ‘^15^School of Health, Fudan University, Key Laboratory of Public Health Safety, Ministry of Education, Shanghai, China’. This has now been amended in all versions of the Article.

