# Supplementary material for: Past and future spread of the arbovirus vectors Aedes aegypti and Aedes albopictus
Source: Nat Microbiol. 2019 Mar 4;4(5):854–63. doi: 10.1038/s41564-019-0376-y (PMC6522366; doi:10.1038/s41564-019-0376-y)
Supplement: Supplementary file 1 — Supplementary Notes, Supplementary Figures 1–13, Supplementary Tables 1–9 and Supplementary References. [file 41564_2019_376_MOESM1_ESM.pdf]

nature microbiology

SUPPLEMENTARY INFORMATION
https://doi.org/10.1038/s41564-019-0376-y

In the format provided by the authors and unedited.

Past and future spread of the arbovirus vectors *Aedes aegypti* and *Aedes albopictus*

Moritz U. G. Kraemer[1,2,3,42]*, Robert C. Reiner Jr[4,42], Oliver J. Brady[5,6,42], Jane P. Messina[7,8,42], Marius Gilbert[9,10,42], David M. Pigott[4], Dingdong Yi[11], Kimberly Johnson[4], Lucas Earl[4], Laurie B. Marczak[4], Shreya Shirude[4], Nicole Davis Weaver[4], Donal Bisanzio[12,13], T. Alex Perkins[14], Shengjie Lai[15,16,17], Xin Lu[18,19,20], Peter Jones[21], Giovanini E. Coelho[22], Roberta G. Carvalho[23], Wim Van Bortel[24,25], Cedric Marsboom[26], Guy Hendrickx[26], Francis Schaffner[27], Chester G. Moore[28], Heinrich H. Nax[29], Linus Bengtsson[17,30], Erik Wetter[17,31], Andrew J. Tatem[16,17], John S. Brownstein[2,3], David L. Smith[4], Louis Lambrechts[32], Simon Cauchemez[33], Catherine Linard[9,34], Nuno R. Faria[1], Oliver G. Pybus[1], Thomas W. Scott[35], Qiyong Liu[36,37,38,39], Hongjie Yu[15], G. R. William Wint[1,40], Simon I. Hay[4,43]* and Nick Golding[41,43]*

[1]Department of Zoology, University of Oxford, Oxford, UK. [2]Harvard Medical School, Harvard University, Boston, MA, USA. [3]Boston Children's Hospital, Boston, MA, USA. [4]Institute for Health Metrics and Evaluation, University of Washington, Seattle, WA, USA. [5]Centre for Mathematical Modelling of Infectious Diseases, London School of Hygiene and Tropical Medicine, London, UK. [6]Department of Infectious Disease Epidemiology, London School of Hygiene and Tropical Medicine, London, UK. [7]School of Geography and the Environment, University of Oxford, Oxford, UK. [8]Oxford School of Global and Area Studies, University of Oxford, Oxford, UK. [9]Spatial Epidemiology Lab (SpELL), Universite Libre de Bruxelles, Brussels, Belgium. [10]Fonds National de la Recherche Scientifique, Brussels, Belgium. [11]Department of Statistics, Harvard University, Cambridge, MA, USA. [12]RTI International, Washington, DC, USA. [13]Epidemiology and Public Health Division, School of Medicine, University of Nottingham, Nottingham, UK. [14]Department of Biological Sciences and Eck Institute for Global Health, University of Notre Dame, Notre Dame, IN, USA. [15]School of Health, Fudan University, Key Laboratory of Public Health Safety, Ministry of Education, Shanghai, China. [16]Department of Geography and Environment, University of Southampton, Southampton, UK. [17]Flowminder Foundation, Stockholm, Sweden. [18]School of Business, Central South University, Changsha, China. [19]College of Systems Engineering, National University of Defense Technology, Changsha, China. [20]School of Business Administration, Southwestern University of Finance and Economics, Chengdu, China. [21]Waen Associates Ltd, Y Waen, Islaw'r Dref, Dolgellau, Gwynedd, UK. [22]Pan American Health Organization (PAHO), Washington, DC, USA. [23]National Dengue Control Program, Ministry of Health, Brasilia, Brazil. [24]European Centre for Disease Prevention and Control, Stockholm, Sweden. [25]Institute of Tropical Medicine, Antwerp, Belgium. [26]Avia-GIS, Zoersel, Belgium. [27]Francis Schaffner Consultancy, Riehen, Switzerland. [28]Department of Microbiology, Immunology, and Pathology, Colorado State University, Fort Collins, CO, USA. [29]Computational Social Science, ETH Zurich, Zurich, Switzerland. [30]Department of Public Health Sciences, Karolinska Institutet, Stockholm, Sweden. [31]Stockholm School of Economics, Stockholm, Sweden. [32]Insect–Virus Interactions Unit, Institut Pasteur, CNRS, UMR2000 Paris, France. [33]Mathematical Modelling of Infectious Diseases Unit, Institut Pasteur, CNRS, UMR2000 Paris, France. [34]Department of Geography, Universite de Namur, Namur, Belgium. [35]Department of Entomology and Nematology, University of California, Davis, Davis, CA, USA. [36]State Key Laboratory of Infectious Disease Prevention and Control, Collaborative Innovation Center for Diagnosis and Treatment of Infectious Diseases, National Institute for Communicable Disease Control and Prevention, Chinese Center for Disease Control and Prevention, Changping, Beijing, China. [37]Shandong University Climate Change and Health Center, School of Public Health, Shandong University, Jinan, Shandong, China. [38]WHO Collaborating Centre for Vector Surveillance and Management, Beijing, China. [39]Chongqing Centre for Disease Control and Prevention, Chongqing, China. [40]Environmental Research Group Oxford (ERGO), Department of Zoology, Oxford University, Oxford, UK. [41]School of BioSciences, University of Melbourne, Parkville, Victoria, Australia. [42]These authors contributed equally: Moritz U. G. Kraemer, Robert C. Reiner Jr, Oliver J. Brady, Jane P. Messina, Marius Gilbert. [43]These authors jointly supervised this work: Simon I. Hay, Nick Golding. *e-mail: moritz.kraemer@zoo.ox.ac.uk; sihay@uw.edu; nick.golding.research@gmail.com

Supplementary Notes

Model validation results

In areas where there is time-series data and empirical human movement data available such as the United States we find that great-circle distance is capturing the spread process of the vector with a univariate correlation between origin of continental spread in the southern United States around 1995 (FIPS: 4019 = Pima Arizona, 4021 = Pinal Arizona, 48061 = Cameron Texas, 48215 = Hidalgo Texas) and distance to them of 0.24 (Pearson correlation, CI: 0.21 – 0.27, p-value: <0.01). In the USA a total 177 out of 3,143 districts (5%) are currently reporting the presence of *Ae. aegypti* mosquitoes. However, this is a very conservative estimate as not all counties carry out routine vector surveillance[1]. *Aedes albopictus* is much more widely distributed in the United States with 37% of all counties reporting presence of the mosquito (1,165 of 3,143 districts). For this second species distance is a stronger indicator of timing of infestation from the first reported mosquito (Houston Texas, 1985) with a Pearson correlation coefficient of 0.40 (CI: 0.37 – 0.43, p-value: <0.01. In Europe, a total of 225 districts out of 1,588 (14%) are currently reporting presence of *Ae. albopictus*. From the origin of the continental spread in Durres, Albania, the vector spread extensively in southern Europe with occasional importations into northern France and Germany. Great-circle distance to the origin in Albania is a strong predictor of risk of spread (Pearson correlation coefficient 0.54, CI: 0.44 – 0.63, p-value: <0.01).

Using a comprehensive probabilistic geographic spread model we reconstructed the spatial spread process, specifically the timing and likelihood of the vectors persistence. For *Ae. aegypti,* in addition to great-circle distance, county-to-county commuting trips, and mobility

metrics were strong predictors reconstructing the expansion in the USA (2006-2016). The predictive power of the model is evaluated against the ability to anticipate the spread of the vector using a training (2006 – 2012) and a test dataset (2013 – 2016) resulting in a mean AUC = 0.80 (95% CI: 0.75 – 0.85, Supplementary Figure 6). Similarly, reconstructing the spread of *Ae. albopictus* from 1986 to 2016 in the United States we find our model to be highly predictive in terms of discriminating the areas with high risk of infestation from those that are at low risk (Supplementary Figure 6). Mean AUC using a training dataset (1986-2005) and testing dataset (2006-2016) is 0.76 (95% CI: 0.66 – 0.87, Supplementary Figure 6). Covariates best explaining the spatial spread are direct neighbour adjacency, great-circle distance, radiation, and gravity models. We repeated the analysis for *Ae. albopictus* in Europe and also show robust predictive ability (mean AUC = 0.90, 95% CI: 0.88 – 0.93, Supplementary Figure 6). Covariates retained in the model were the same for both models except the greater risk of infestation when a district was two degrees away from an infested district.

The environmental niche modelling was performed for both species. The 2015 baseline model using environmental variables and species occurrence data resulted in high predictive power for *Ae. aegypti* (AUC = 0.865, 95% CI = 0.85 – 0.87) and *Ae. albopictus* (AUC = 0.90, 95% CI = 0.88 – 0.91). Variables that explained most of the variation of the species environmental niche was the species-specific temperature suitability indices (58% for *Ae. aegypti* and 49% for *Ae. albopictus*) followed by maximum precipitation (16% for *Ae. aegypti* and 19% for *Ae. albopictus*). A full list is available in Table 1 and 2.

Comparison to previously published work and CDC and ECDC records

Aedes albopictus in Europe: Overall, there is a high concordance between the current reported distribution of *Ae. albopictus* and the present day predicted distribution as determined by our model. Both suggest that the primary range of *Ae. albopictus* is concentrated around the Mediterranean coastline with extensions across southern France. Our model-based predictions also suggest a high probability of presence in Bulgaria, Romania and Hungary, regions for which ECDC data is patchy or missing entirely. Furthermore, we predict relatively low levels of suitability for areas for which ECDC has observed introduction but no subsequent establishment, such as central France, southern Germany and southern England. One noticeable discrepancy is the reported absence of *Ae. albopictus* in Portugal, a country we predict to be both highly suitable and highly connected to the species' established range. This may be due to the biogeographical barrier of the arid interior of the Iberian peninsula which may desiccate eggs and juveniles in transit.

Aedes aegypti in Europe: There is a high degree of consensus between the reported absence of *Ae. aegypti* across Europe and the 2015 predicted distribution from our model. While we do predict low probability of presence in some regions of the southern Mediterranean (Sicily and Lebanon) which have not yet reported *Ae. aegypti* this remains only a minor discrepancy. The biggest inconsistency is in the Eastern black sea region where established *Ae. aegypti* populations have been reported to occur over multiple years, yet our model predicts negligible risk for these regions. Therefore, our model may under predict risk in far south Eastern Europe.

Aedes albopictus in the USA: There is a high degree of concordance between predicted and observed *Ae. albopictus* distribution in the USA as of 2015 with the majority of Eastern and Mid-Western States reporting presence of the vector. The model also correctly identifies

patchy incursions in the south western states, however, there is slight overestimation of risk (when comparing to reported occurrences) in the northern states (e.g. South Dakota and Wisconsin).

Aedes aegypti in the USA: Our model predictions for *Ae. aegypti* in the USA in 2015 are largely consistent with reported occurrences of the species with the most consistent presence in Florida, a northern limit of around Washington DC, a patchy distribution in Texas and slight incursion into California. Discrepancies do exist in many southern states, however, where the model predicts widespread presence but only sporadic records of presence have been reported. Given reported presence of the species on the northern, southern and western borders of these regions, it remains unclear if these absences reflect true absences or absences in reporting.

A PubMed search for *Aedes* AND future AND [global OR Europe OR USA] returned 108, 55, and 131 results respectively which were distilled to the 16 references in Supplementary Table 9 based on the inclusion criteria: i) gave projections of the future distribution of either species and ii) made predictions at the scale of whole country or US state or higher.

Methodologically, past approaches to project the future distribution of *Aedes* have largely fallen into two camps: i) agent based, mechanistic dynamic models or ii) species distribution models using the Maximum Entropy (MAXENT) algorithm. These methods, in isolation, are only suitable for estimating suitability for *Aedes* populations and do not attempt to estimate whether it would be possible for any *Aedes* species to spread into this newfound niche. In addition, the Boosted Regression Tree species distribution model used here presents a considerable advantage over the MAXENT algorithm for this application

due to its more explicit handling of biases in reporting [2], a key feature of spatially variable *Aedes* surveillance.

All previous modelling attempts also only consider changes in climatological factors, principally temperature and precipitation (Supplementary Table 9). Previous mapping studies have shown that the current global distribution of *Ae. aegypti* and *Ae. albopictus* is also determined by socioeconomic factors such as urbanization[3]. Given the importance of these variables, we have included them in our assessment of the future distribution of these mosquito species.

In Europe, the majority of these climatological projection models suggest *Ae. albopictus* will spread through France to cover much of western Europe and southeast England with some suggesting extensions as far north as southern Sweden. In contrast, our own mapping efforts suggest much more limited spread over this time period with modest increases in range along the fringes of the European distribution of *Ae. albopictus*.

In the USA, previous predictions for *Ae. albopictus* suggest the species will spread throughout the country by 2080 with the exception of the arid southern state of Arizona and the northern states of north Dakota and Wisconsin[4,5], while much of the south east of the USA from Texas to Virginia is predicted to be suitable for *Ae. aegypti* at least for some times of the year[6].

For *Ae. aegypti* in the USA our predictions are similar to previous efforts, however we do predict further spread to some major cities in the northeast and inland California that previous models have not. For *Ae. albopictus* while our estimates agree on the northern

limit of the species with those from previous approaches, we predict that the species' distribution will be largely restricted East of the Rocky Mountains.

Elsewhere, all past approaches agree that there will be considerable change in suitability in Asia (in particular India, Thailand and Vietnam) for both species with both increases and decreases in suitability, but there is a lack of consensus on which areas will see expansion or contraction. Our predictions of modest changes across the continent are in line with previous findings.

The combination of more advanced methodological approaches with more detailed covariates helps explain much of the difference between past projections and those presented here, in particular the inclusion of socioeconomic as well as climatic factors.

Supplementary Figures

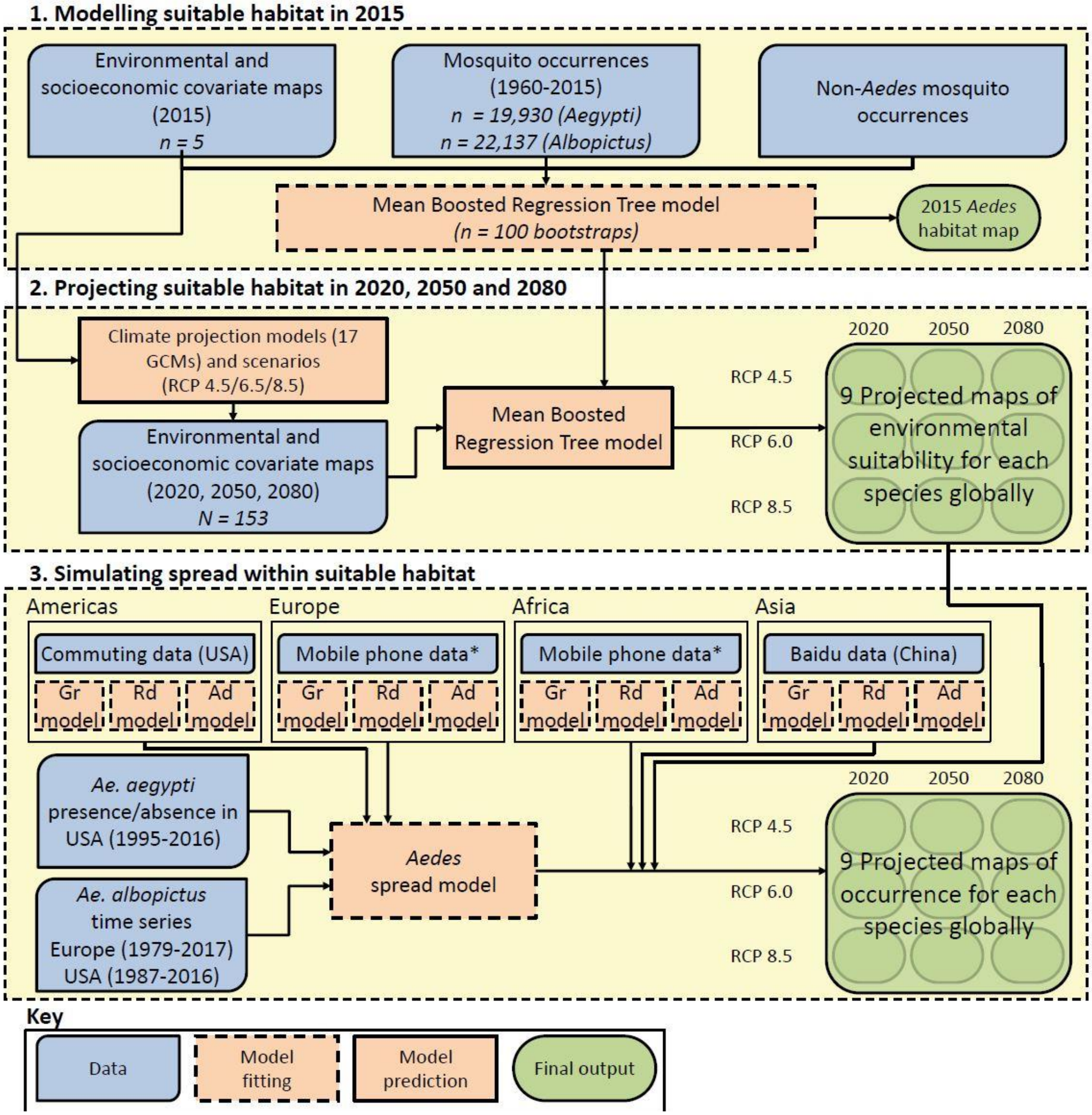

Supplementary Figure 1: Full description of data and methods used to predict the geographic spread of *Aedes aegypti* and *Aedes albopictus* in 2020, 2050 and 2080.

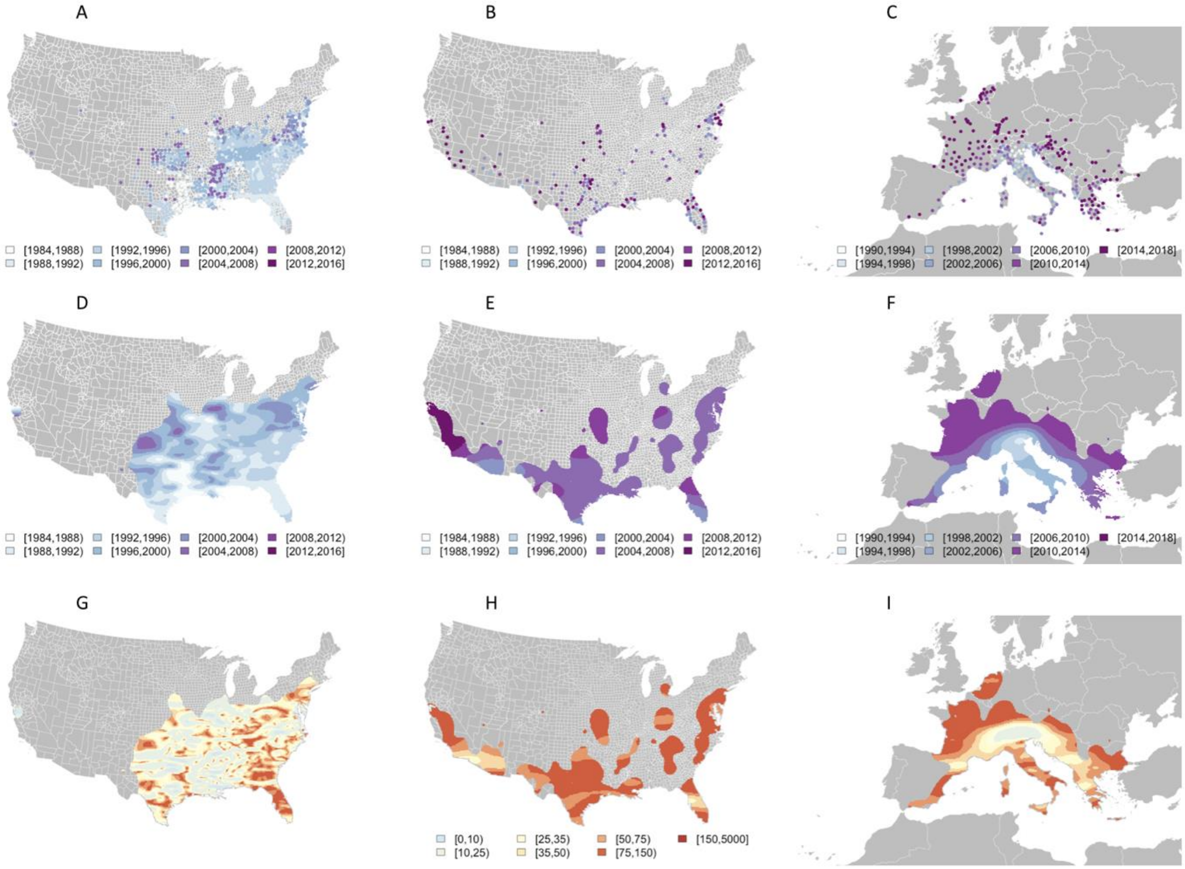

Supplementary Figure 2: Data for model fitting and evaluation. Dates of observations per county for *Aedes albopictus* (A, C) where white is the earliest detection and purple the latest. B shows the observations per county in the United States of America (USA) for *Aedes aegypti*. D - F show the interpolated earliest detection at a 10 km resolution grid for both species and the USA (D, E) and Europe (F). G-I show the estimated spread rates in km per year based on a thin plate spline regression (TPSR) with kernel density smoothing of 100 km.

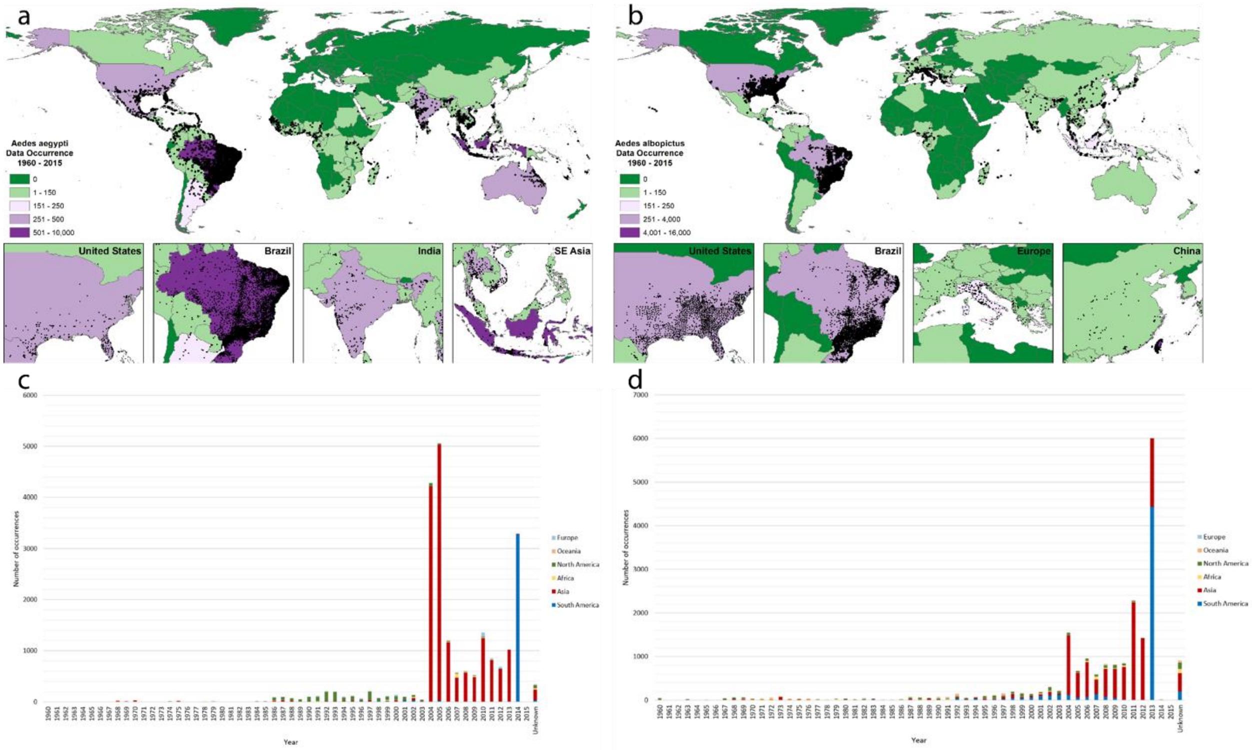

Supplementary Figure 3: Maps showing the spatial global distribution of occurrence data of Aedes aegypti (a) and Ae. albopictus (b). Each black dot corresponds to an occurrence and colours represent the number of occurrences. The temporal distribution for *Ae. aegypti* (c) and *Ae. albopictus* (d) with different colours representing different continents.

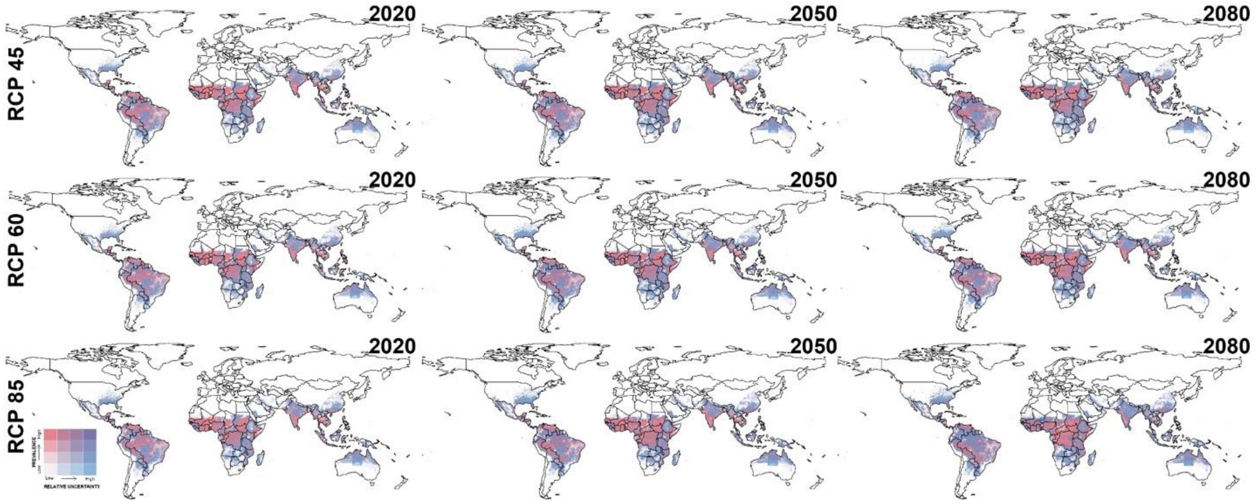

Supplementary Figure 4: Global maps of the predicted spread of *Ae. aegypti* globally for all climatic scenarios (RCP 4.5, RCP 6.0, and RCP 8.5) in 2020, 2050 and 2080. Predicted suitability of Ae. *aegypti* quantile cutoff points were 0.24, 0.66, 0.88. Relative uncertainty was computed as the ratio of the 95% uncertainty intervals and predicted *Ae. aegypti* suitability for each pixel. Cutoff points for uncertainty were 0.08, 0.18, 0.31. The lowest quantile of predicted suitability is shown in white, and the highest in dark pink. The lowest quantile for uncertainty is white and the highest is blue. The colours overlap such that areas coloured purple have both high predicted suitability of *Ae. aegypti* and high relative uncertainty. Pixels with no predicted suitability are coloured in grey.

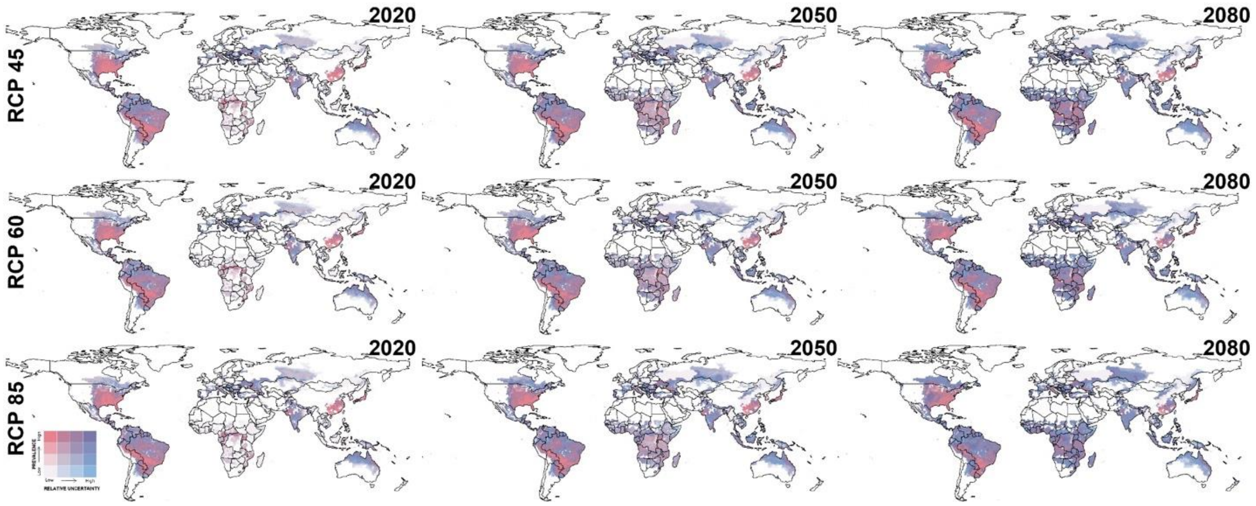

Supplementary Figure 5: Global maps of the predicted spread of *Ae. albopictus* globally for all climatic scenarios (RCP 4.5, RCP 6.0, and RCP 8.5) in 2020, 2050 and 2080. Predicted suitability of Ae. *albopictus* quantile cutoff points were 0.13, 0.41, 0.70. Relative uncertainty was computed as the ratio of the 95% uncertainty intervals and predicted *Ae. albopictus* suitability for each pixel. Cutoff points for uncertainty were 0.16, 0.36, 0.53. The lowest quantile of predicted suitability is shown in white, and the highest in dark pink. The lowest quantile for uncertainty is white and the highest is blue. The colours overlap such that areas coloured purple have both high predicted suitability of *Ae. albopictus* and high relative uncertainty. Pixels with no predicted suitability are coloured in grey.

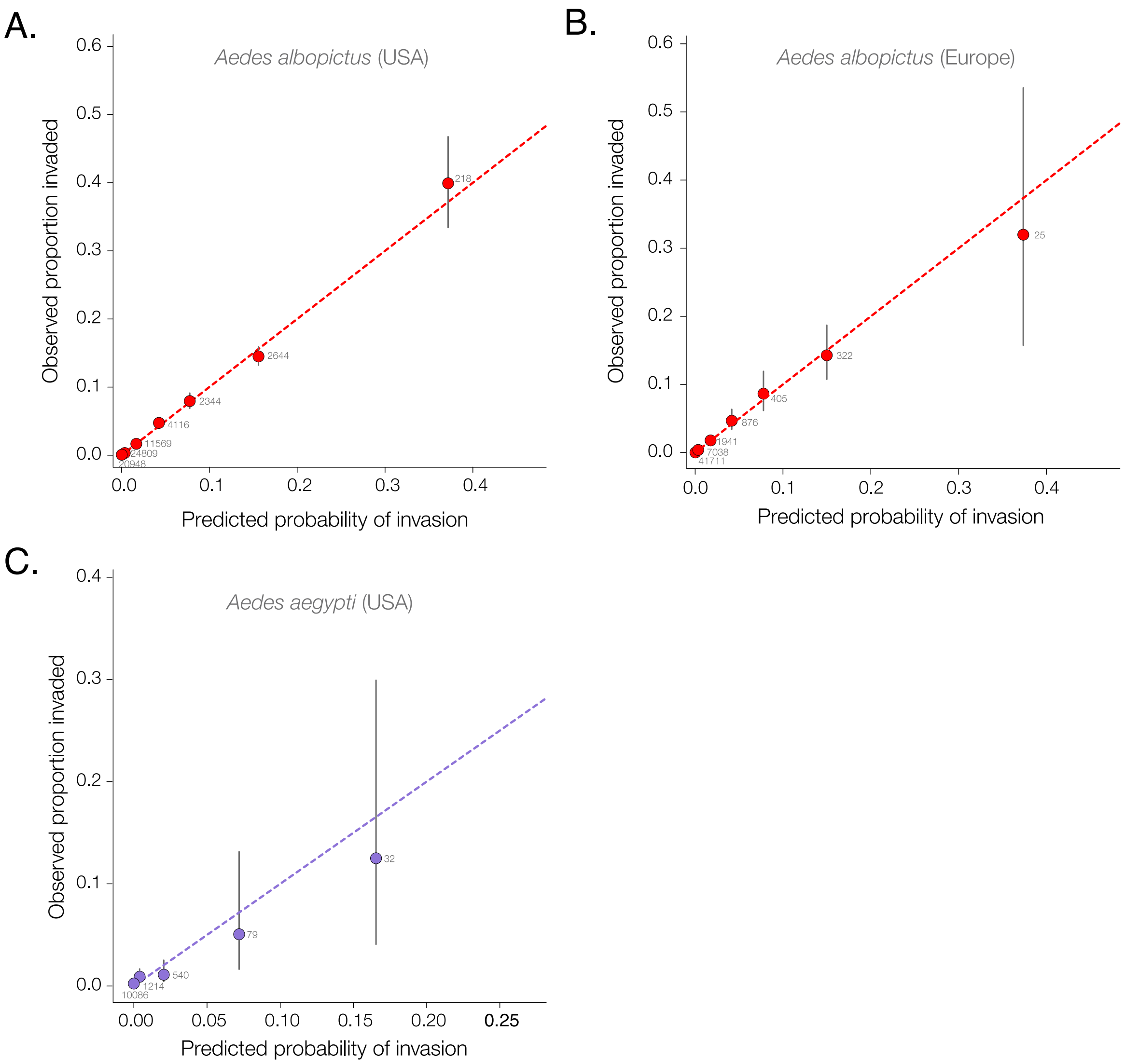

Supplementary Figure 6: Evaluation of geographic spread model showing the predicted probability of infestation compared to infestation that occurred in Europe and the United States. Models were run on subsets of data and predicted probabilities were compared to new invasions (out of sample) at the later stage of the spread process. Groupings for *Ae. albopictus* were [0.001;0.01,0.03,0.06,0.1,0.3,0.5,0.7, 1] and for *Ae. aegypti* [0.001,0.01,0.05,0.1, 1] where the dots represent the mean predicted probability for each group. The number indicates the number of predicted and observed proportion falling within each group. The grey line represents the range of values within each of the groups. The dotted line shows the perfect correspondence between predicted vs. observed spread.

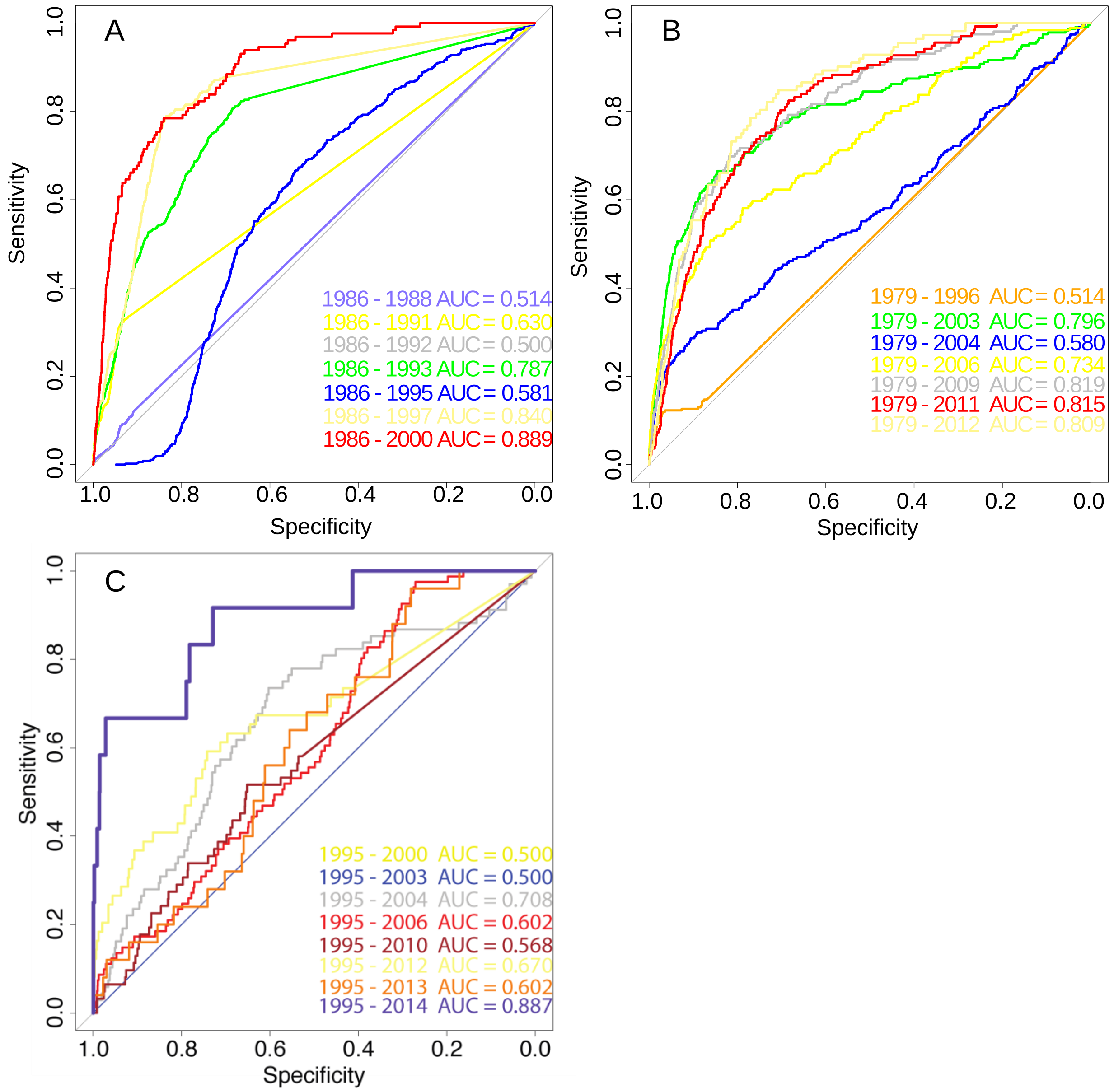

Supplementary Figure 7: Out of sample predictive accuracy as measured by Area Under the Curve (AUC) for *Ae. albopictus* in the United States (A*) Ae. albopictus* in Europe (B) and *Ae. aegypti* in the United States (C). Different colours represent different training datasets. Evaluation was always done on the subsequent set of data going forward to the last year of data.

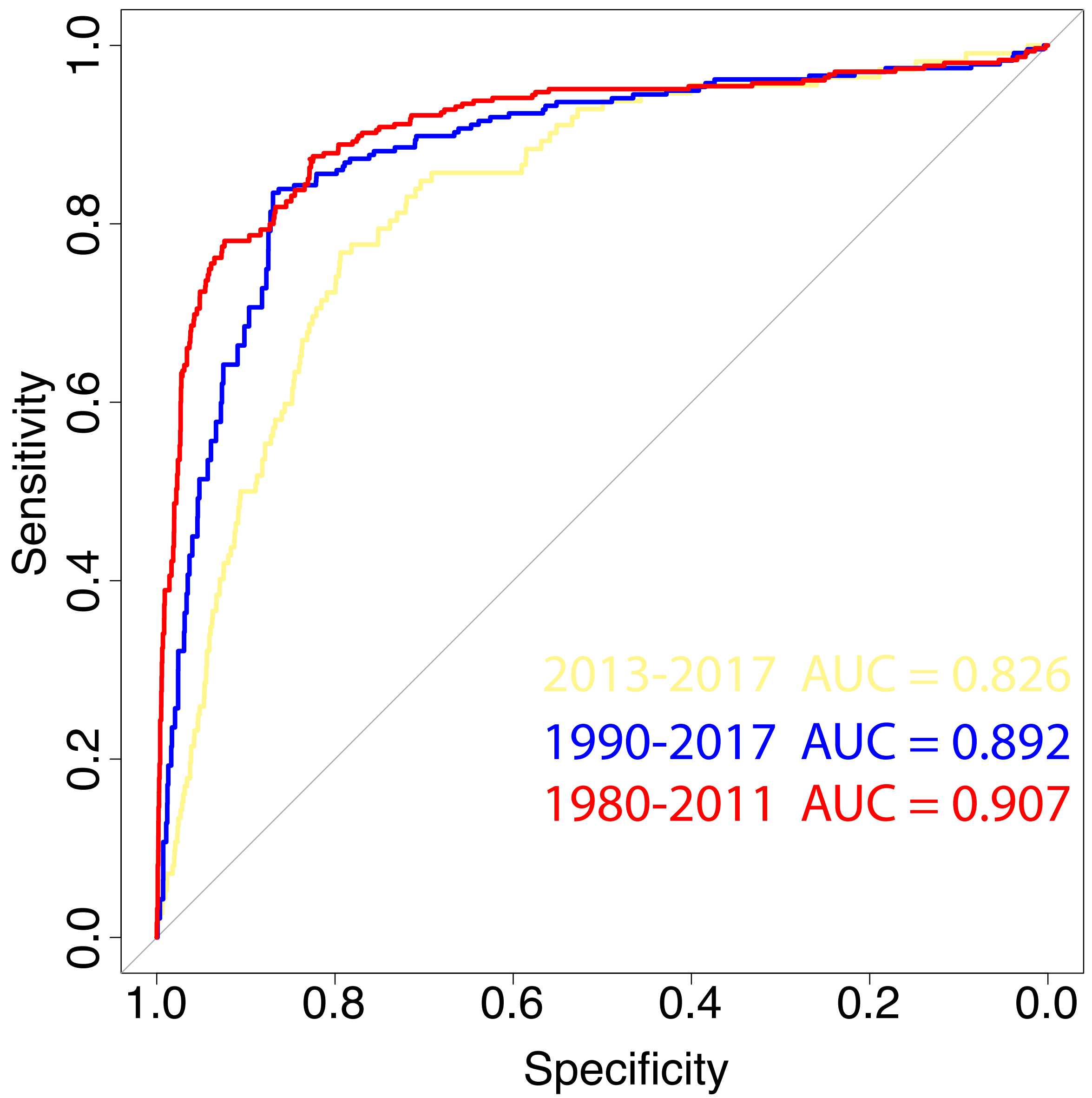

Supplementary Figure 8: Out of sample prediction of a model fitted to USA occurrence data for *Ae. albopictus* and applied to Europe. Different lines represent different periods of evaluation on the European *Ae. albopictus* data.

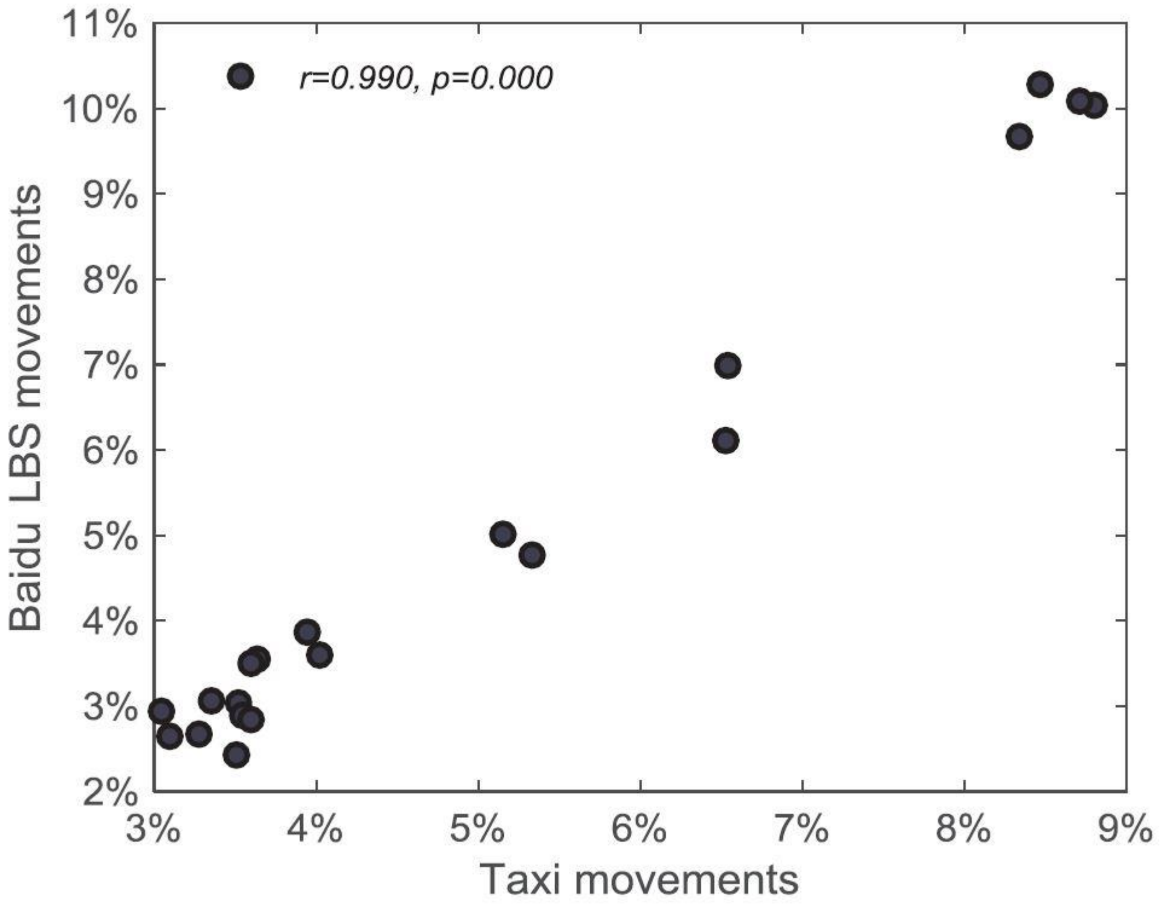

Supplementary Figure 9: Comparison of movements extracted from Taxi's GPS and from Baidu LBS. Each data point represents the proportion of movements between each pair of districts. The correlation between Taxi movements and Baidu movements Pearson's r = 0.99.

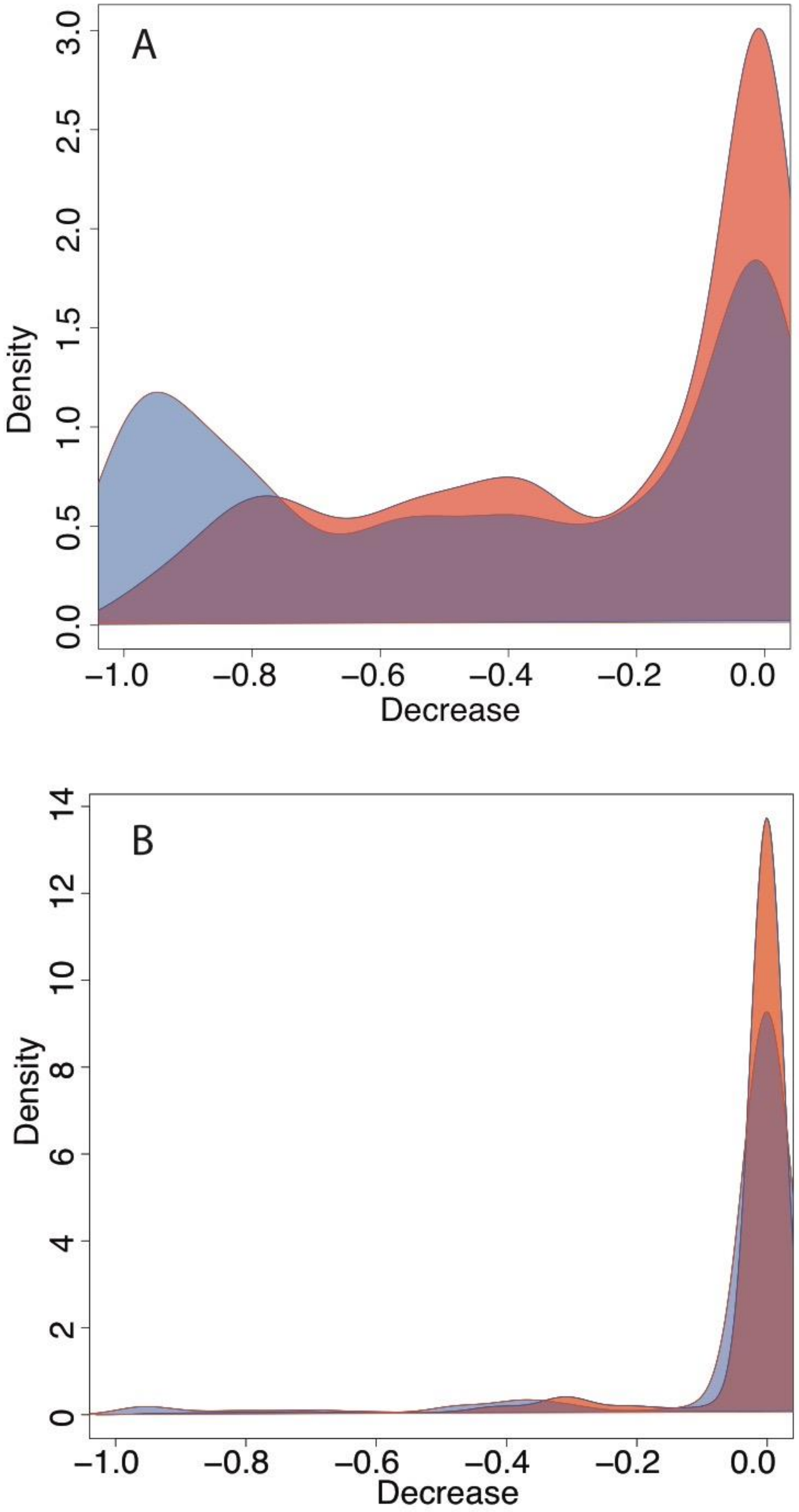

A
Density
3.0
2.5
2.0
1.5
1.0
0.5
0.0
−1.0
−0.8
−0.6
−0.4
−0.2
0.0
Decrease
B
Density
14
12
10
8
6
4
2
0
−1.0
−0.8
−0.6
−0.4
−0.2
0.0
Decrease

Supplementary Figure 10: Comparing predictions of *Ae. aegypti* (A) and *Ae. albopictus* (B) for the United States when limiting the potential for spread using human mobility metrics and distance metrics to 200km vs. 1000km for 2020 (blue) and 2050 (red).

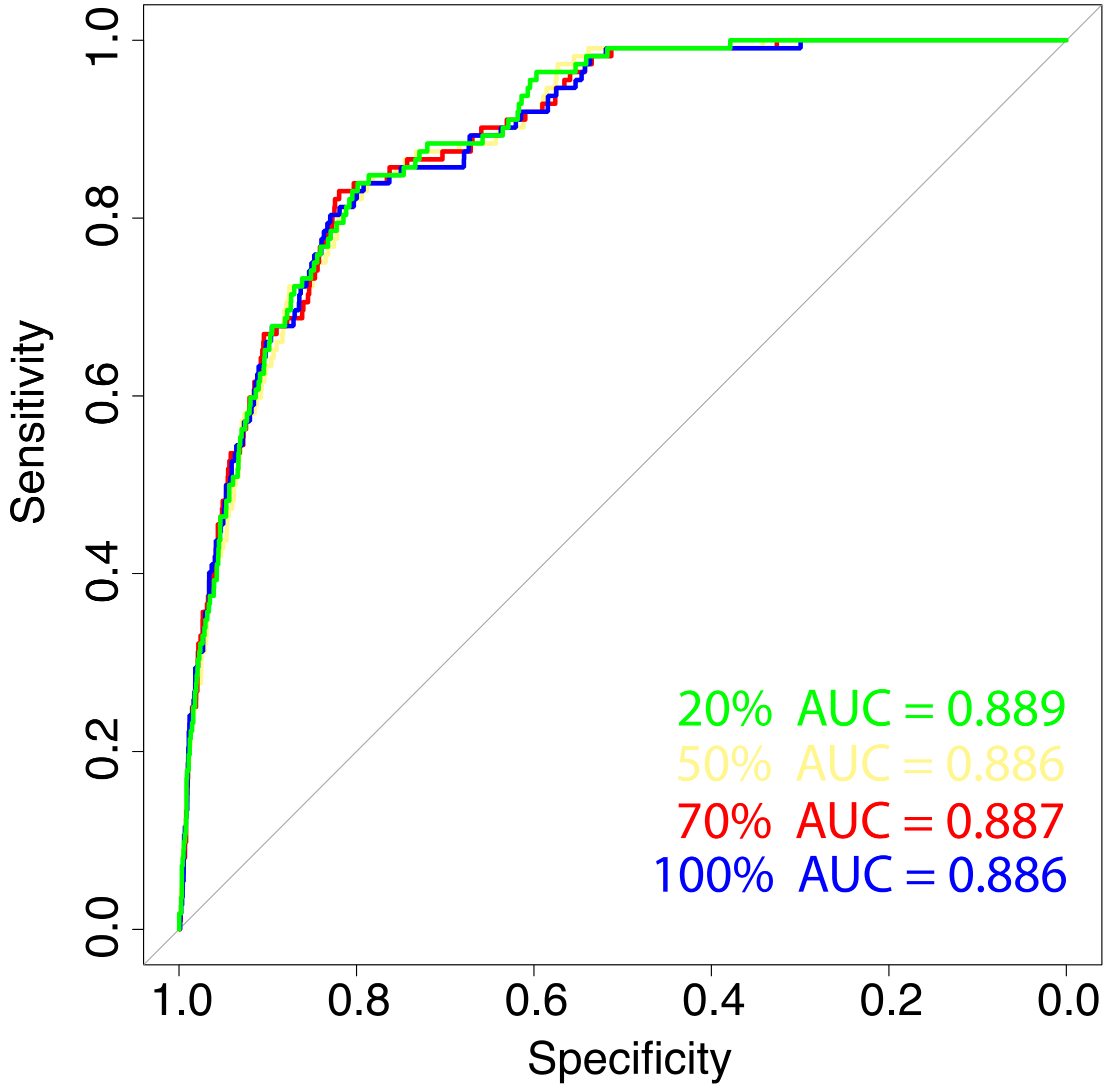

Supplementary Figure 11: Predictive accuracy when decreasing movements between countries to within country movement to predict the spread of *Ae. albopictus* in Europe from 2014-2017 (fitted between 1979-2013). Green shows a reduction in between country movement of 80% (Area Under the Curve (AUC) = 0.889), khaki 50% (AUC = 0.886), red 30% (AUC = 0.887) and blue (AUC = 0.885) is baseline.

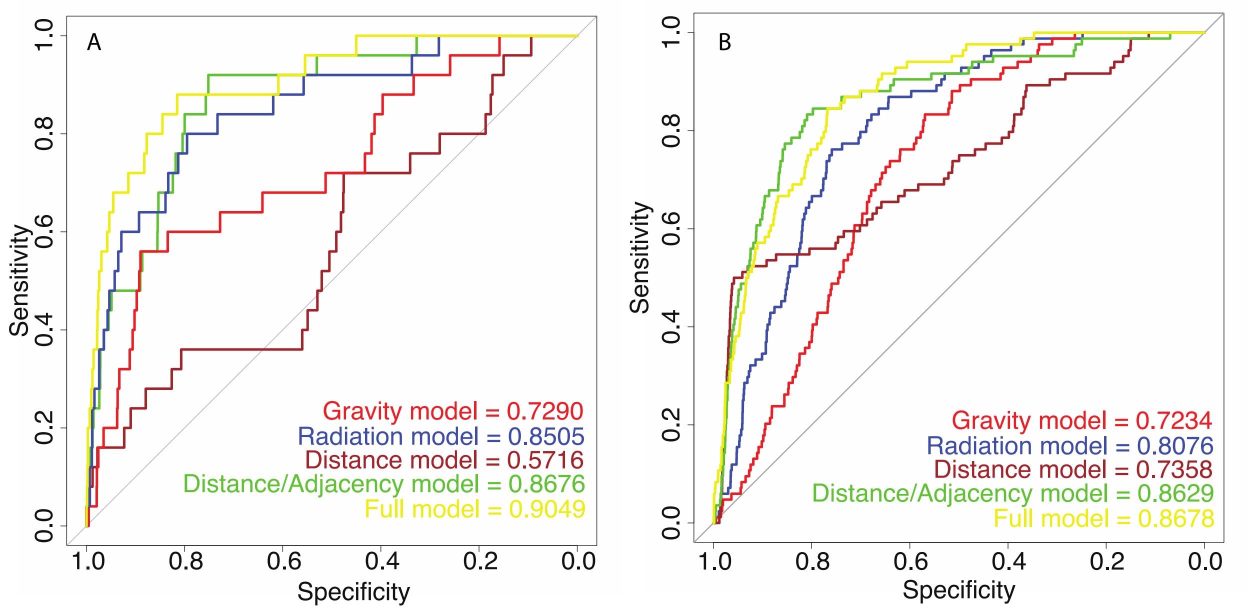

Supplementary Figure 12: Out of sample prediction of *Aedes aegypti (A)* spread in the United States comparing single variable models to the full model (training 1986-2002, testing: 2003-2013). B shows out of sample predictions for *Ae. albopictus* in the United States (training: 1995-2010, testing: 2011-2016).

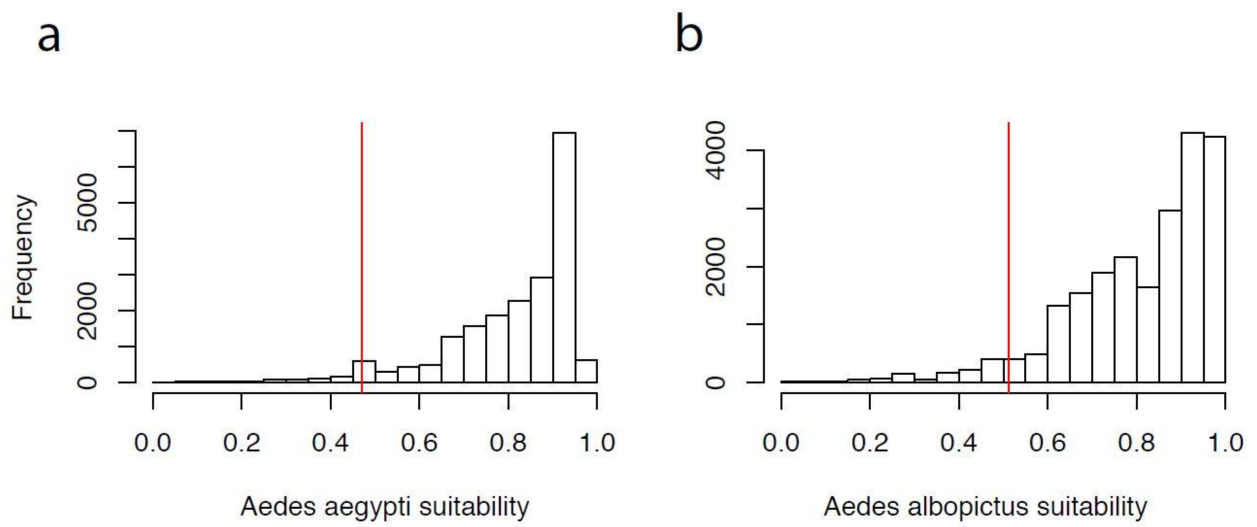

Supplementary Figure 13: Histograms of predicted suitability for locations of observations. This data was used to derive cut-offs to build binary presence and absence maps for *Ae. aegypti* (a) and *Ae. albopictus* (b) species so the relevant population at risk and area expansion could be calculated.

Supplementary Tables:

	mean	2.50%	97.50%
Temperature suitability index (0-1)	58.46	57.09	59.55
Maximum precipitation (mm)	16.22	15.27	17.26
Relative humidity (%)	10.11	9.41	10.97
Minimum precipitation (mm)	9.47	8.72	10.12
Probability urban (%)	5.74	5.26	6.40

Supplementary Table 1: Relative contribution of each variable used in the Boosted Regression Tree Model for Ae. aegypti.

	mean	2.50%	97.50%
Temperature suitability index (0-1)	48.85	47.23	50.04
Maximum precipitation (mm)	18.61	17.56	19.73
Minimum precipitation (mm)	16.58	15.22	17.76
Relative humidity (%)	11.08	10.05	12.13
Probability urban (%)	4.88	4.28	5.38

Supplementary Table 2: Relative contribution of each variable used in the Boosted Regression Tree Model for Ae. albopictus.

Name	Fit data date range	Fit data type	Total number of points*
Model 1	1979-2017	Occurrence and longitudinal	2,704
Model 2	2013-2017	Longitudinal only	344
Model 3	2013-2017	Occurrence and longitudinal	1,111

Supplementary Table 3: Data used to evaluate the spread model in Europe. *Points correspond to unique detections of *Ae. albopictus* in district within one calendar year

Name	Total number of new districts infested 2013-2017 (95% uncertainty interval)
Model 1	22 (15-31)
Model 2	89 (71-105)
Model 3	20 (13-28)

Supplementary Table 4: Simulated spread using different sets of models in Europe to contrast the performance under different surveillance efforts.

Species	Continent	Model variant	Total deviance in holdout sets
Ae. albopictus	Europe	Baseline	**21,544**
		Baseline + s(Year)	101,137
	USA	Baseline	**2,876**
		Baseline + s(Year)	3,291
		Baseline + (Year > 2003)	2,904
Ae. aegypti	USA	Baseline	**1045**
		Baseline + s(Year)	2804
		Baseline + (Year > 2003)	1071

Supplementary Table 5: Changes of surveillance activity under different assumptions.

Species	Coefficient (Year > 2003)	Percentage increase in deviance over baseline model
Ae. aegypti	0.83	+2.5%

Ae. albopictus	-1.32	+1.0%

Supplementary Table 6: Evaluation of spread model in the United States before and after the 2003 West Nile Virus outbreak.

		Model	Institution	Resolution, Lat/Long	Reference	Replicates RCP Hist*	Replicates RCP 4.5	Replicates RCP 6.0	Replicates RCP 8.5
1		BCC-CSM 1.1	Beijing Climate Centre, China Meteorological Administration	2.8125 x 2.8125	[7]	3	1	1	1
2		BCC-CSM 1.1(m)	Beijing Climate Centre, China Meteorological Administration	2.8125 x 2.8125	[7]	3	1	1	1
3		CSIRO-Mk3.6.0	CSIRO and the Queensland Climate Change Centre of Excellence	1.875 x 1.875	[8]	10	10	10	10
		FIO-ESM	The First Institute of Oceanography, SOA, China	2.812 x 2.812	[9]	3	3	3	3
5		GFDL-CM3	Geophysical Fluid Dynamics Laboratory	2.0 x 2.5	[10]	5	1	1	1
6		GFDL-ESM2G	Geophysical Fluid Dynamics Laboratory	2.0 x 2.5	[11]	1	1	1	1

7		GFDL-ESM2M	Geophysical Fluid Dynamics Laboratory	2.0 x 2.5	[11]	1	1	1	1
8		GISS-E2-H	NASA Goddard Institute for Space Studies	2.0 x 2.5	[12]	15	1	3	3
9		GISS-E2-R	NASA Goddard Institute for Space Studies	2.0 x 2.5	[12]	10	7	3	3
10		HadGEM2-ES	Met Office Hadley Centre	1.2414 x 1.875	[13]	4	4	4	4
11		IPSL-CM5A-LR	Institut Pierre-Simon Laplace	1.875 x 3.75	[14]	6	4	1	1
12		IPSL-CM5A-MR	Institut Pierre-Simon Laplace	1.2587 x 2.5	[14]	3	1	1	1
13		MIROC-ESM	Atmosphere and Ocean Research Institute (The University of Tokyo), National Institute for Environmental Studies, and Japan Agency for Marine-Earth Science and Technology	2.8125 x 2.8125	[15]	5	3	1	3
14		MIROC-ESM-CHEM	Atmosphere and Ocean Research Institute (The	2.8125 x 2.8125	[15]	3	1	1	1

			University of Tokyo), National Institute for Environmental Studies, and Japan Agency for Marine-Earth Science and Technology						
15		MIROC5	Japan Agency for Marine-Earth Science and Technology, Atmosphere and Ocean Research Institute (The University of Tokyo), and National Institute for Environmental Studies	1.4063 x 1.4063	[16]	1	1	1	1
16		MRI-CGCM3	Meteorological Research Institute	1.125 x 1.125	[17]	5	1	1	1
17		NorESM1-M	Norwegian Climate Centre	1.875 x 2.5	[18,19]	3	1	1	1

Supplementary Table 7: Details of 17 Global Climate Model ensembles used for the study. *Hist is the number of runs of historic data for baseline evaluation

year	RCP	Ae. aegypti, # of countries	Ae. aegypti, country names	Ae. albopictus # of countries	Ae. albopictus; country names
2015	baseline	156	NA	177	NA
2020	4.5	155	NA	180	Jersey, Chad, Comoros
2050	4.5	156	Swaziland	180	Jersey, Chad, Comoros
2080	4.5	156	NA	181	Belgium, Jersey, Chad, Solomon Islands
2020	6	157	Swaziland	191	Poland, Netherlands, Germany, Belgium, Czech Republic, Jersey, Mali, Chad, Burkina Faso, Solomon Islands, Comoros Samoa, Saint Helena, Europa Island
2050	6	158	Saint Helena, Swaziland	191	Poland, Netherlands, Belgium, Germany, Jersey, Czech Republic, Mali, Chad, Burkina Faso, Solomon Islands, Comoros, Samoa, Saint Helena, Europa Island
2080	6	159	Canada, Saint Helena, Swaziland	197	Norway, Poland, Germany, Belarus, Netherlands, U.K. of Great Britain and Northern Ireland, Belgium, Czech Republic, Jersey, Bahrain, Saint Kitts and Nevis, Montserrat, Mali, Chad, Burkina Faso, Solomon Islands, Comoros, Samoa Saint Helena, Europa Island
2020	8.5	159	Canada, Saint Helena, Swaziland	195	Norway, Lithuania, Poland, Belarus, Germany, Netherlands, Belgium, Czech Republic, Jersey, Saint Kitts and Nevis, Montserrat, Mali, Burkina Faso, Chad, Solomon Islands, Comoros, Samoa, Saint Helena
2050	8.5	159	Canada, Saint Helena, Swaziland	197	Norway, Denmark, Poland, U.K. of Great Britain and Northern Ireland, Germany, Belarus, Netherlands, Belgium, Czech Republic, Jersey, Saint Kitts and Nevis, Montserrat, Mali, Burkina Faso, Chad, Solomon Islands, Comoros, Samoa Saint Helena, Europa Island
2080	8.5	162	Canada, Croatia, Greece, Cayman Islands, Saint Helena, Swaziland	209	Norway, Finland, Estonia, Sweden, Latvia, U.K. of Great Britain and Northern Ireland, Denmark, Lithuania, Belarus, Germany, Poland, Netherlands, Ireland, Belgium, Czech Republic, Mongolia, Luxembourg, Guernsey, Jersey, Liechtenstein, Kuril islands, Andorra, Bahrain, Saint Kitts and Nevis, Montserrat, Mali, Burkina Faso, Chad, Solomon Islands, Comoros, Samoa, Saint Helena, Europa Island

Supplementary Table 8: Number of countries expected to report Ae. aegypti and Ae. albopictus for each scenario and from 2015 to 2080.

Reference	Species	Modelling approach	Covariates included
**Europe**			
Cunze et al 2016[20]	*Albopictus*	MAXENT species distribution model	Temperature and precipitation
Cunze et al 2016[21]	*Albopictus*	MAXENT species distribution model	Temperature, precipitation and photoperiod
Liu Helmersson et al 2016[22]	*Aegypti and Albopictus*	Mechanistic model of vectorial capacity	Temperature
Koch et al 2015[23]	*Albopictus*	MAXENT species distribution model	Temperature and precipitation
Fischer et al 2013[24]	*Albopictus*	MAXENT species distribution model	Temperature, precipitation and altitude
Caminade et al 2012[25]	*Albopictus*	Mechanistic model of climatic limits	Temperature, precipitation
ECDC 2009[26]	*Albopictus*	Mechanistic model of climatic limits	Temperature and Precipitation
**USA**			
Butterworth et al 2017[6]	*Aegypti*	Mechanistic model of mosquito population dynamics	Temperature and precipitation
Rochlin et al 2013[4]	*Albopictus*	MAXENT species distribution model	Temperature, precipitation and land use
Ogden et al 2014[5]	*Albopictus*	Mechanistic model of climatic limits	Temperature
**Global**			

Campbell et al 2015[27]	*Aegypti and Albopictus*	MAXENT species distribution model	Temperature and precipitation
Proestos et al 2015[28]	*Albopictus*	Mechanistic model of climatic limits	Temperature and precipitation
Khormi and Kumar 2014[29]	*Aegypti*	Mechanistic model of climatic limits	Temperature, precipitation and humidity
Capinha et al 2014[30]	*Aegypti*	Alpha-shapes species distribution model	Temperature and precipitation
Liu Helmersson et al 2014[31]	*Aegypti*	Mechanistic model of vectorial capacity	Temperature

Supplementary Table 9: Past published projections of the distribution of *Ae.aegypti* and *Ae. albopictus*

Supplementary References

1. Hahn, M. B. *et al.* Reported Distribution of Aedes (Stegomyia) aegypti and Aedes (Stegomyia) albopictus in the United States, 1995-2016 (Diptera: Culicidae). *J. Med. Entomol.* 1–7 (2016). doi:10.1093/jme/tjw072
2. Elith, J., Leathwick, J. R. & Hastie, T. A working guide to boosted regression trees. *J. Anim. Ecol.* **77**, 802–13 (2008).
3. Kraemer, M. U. G. *et al.* The global distribution of the arbovirus vectors Aedes aegypti and Ae. albopictus. *eLife* **4**, e08347 (2015).
4. Rochlin, I., Ninivaggi, D. V, Hutchinson, M. L. & Farajollahi, A. Climate Change and Range Expansion of the Asian Tiger Mosquito (Aedes albopictus) in Northeastern USA : Implications for Public Health Practitioners. *PLoS ONE* **8**, e60874 (2013).
5. Ogden, N. H., Milka, R., Caminade, C. & Gachon, P. Recent and projected future climatic suitability of North America for the Asian tiger mosquito Aedes albopictus. *Parasit. Vectors* **7**, in press (2014).
6. Butterworth, M. K., Morin, C. W. & Comrie, A. C. An Analysis of the Potential Impact of Climate Change on Dengue Transmission in the Southeastern United States. *Environ. Health Perspect.* **125**, 579–585 (2017).
7. Wu, T. A mass-flux cumulus parameterization scheme for large-scale models: description and test with observations. *Clim Dyn* **38**, 725–744 (2012).
8. Collier, M. A. *et al.* The CSIRO-Mk3.6.0 Atmosphere-Ocean GCM: participation in CMIP5 and data publication. in *19th International Congress on Modelling and Simulation* (2011).
9. Song, Z., Qiao, F. & Song, Y. Response of the equatorial basin-wide SST to non-breaking surface wave-induced mixing in a climate model : An amendment to ... (2015). doi:10.1029/2012JC007931

10. Donner, L. J., Wyman, B. L., Hemler, R. S., Horowitz, L. W. & Ming, Y. The Dynamical Core, Physical Parameterizations, and Basic Simulation Characteristics of the Atmospheric Component AM3 of the GFDL Global Coupled Model CM3. *J. Clim.* **24**, 3484–3519 (2011).
11. Dunne, J. P., John, J. G., Adcroft, A. J. & Griffies, S. M. GFDL's ESM2 Global Coupled Climate – Carbon Earth System Models. Part I: Physical Formulation and Baseline Simulation Characteristics. *J. Clim.* **25**, 6646–6665 (2012).
12. Schmidt, G. A. *et al.* Present-Day Atmospheric Simulations Using GISS ModelE: Comparison to In Situ, Satellite, and Reanalysis Data. *J. Clim.* **19**, 153–192 (2006).
13. Collins, W. J. *et al.* Model Development Development and evaluation of an Earth-System model – HadGEM2. *Geosci. Model Dev.* **4**, 1051–1075 (2011).
14. Dufresne, J.-L. *et al. Climate change projections using the IPSL-CM5 Earth System Model: from CMIP3 to CMIP5*. (2013). doi:10.1007/s00382-012-1636-1
15. Watanabe, S. *et al.* Model Development MIROC-ESM 2010: model description and basic results of CMIP5-20c3m experiments. *Geosci. Model Dev.* **4**, 845–872 (2011).
16. Watanabe, M., Suzuki, T., O'ishi, R., Komuro, Y. & Watanabe, S. Improved Climate Simulation by MIROC5 : Mean States, Variability, and Climate Sensitivity. *J. Clim.* **23**, 6312–6335 (2010).
17. Yukimoto, S., Adachi, Y., Hosaka, M. & Sakami, T. A New Global Climate Model of the Meteorological Research Institute : MRI-CGCM3 — Model Description and Basic Performance —. *J. Meterological Soc. Jpn.* **90A**, 23–64 (2012).
18. Seland, O., Iversen, T., Kirkevag, A. & Storelvmo, T. Aerosol-climate interactions in the CAM-Oslo atmospheric GCM and investigation of associated basic shortcomings. *Tellus* **60A**, 459–491 (2008).
19. Kirkevag, A. *et al.* Aerosol-cloud-climate interactions in the climate model CAM-Oslo. *Tellus* **60A**, 492–512 (2008).

20. Cunze, S., Koch, L. K., Kochmann, J. & Klimpel, S. Aedes albopictus and Aedes japonicus - two invasive mosquito species with different temperature niches in Europe. *Parasit. Vectors* **9**, (2016).

21. Cunze, S., Kochmann, J., Koch, L. K. & Klimpel, S. Aedes albopictus and Its Environmental Limits in Europe. *PLoS ONE* **11**, e0162116 (2016).

22. Liu-helmersson, J., Quam, M., Wilder-smith, A., Stenlund, H. & Ebi, K. EBioMedicine Climate Change and Aedes Vectors: 21st Century Projections for Dengue Transmission in Europe. *EBIOM* **7**, 267–277 (2016).

23. Koch, L. K. *et al.* Modeling the habitat suitability for the arbovirus vector Aedes albopictus (Diptera: Culicidae) in Germany. *Parasitol. Res.* **115**, 957–964 (2016).

24. Fischer, D. *et al.* Climate change effects on Chikungunya transmission in Europe: geospatial analysis of vector's climatic suitability and virus' temperature requirements. *Int. J. Health Geogr.* **12**, 51 (2013).

25. Caminade, C. *et al.* Suitability of European climate for the Asian tiger mosquito Aedes albopictus: recent trends and future scenarios. *J R Soc Interface* **9**, 2708–17 (2012).

26. ECDC, Development of Aedes albopictus risk maps. 2009, European Centres for Disease Control: Stockholm.

27. Campbell, L. P. *et al.* Climate change influences on global vector distributions for dengue and chikungunya viruses. *Phil Trans R Soc B* **370**, 20140135 (2015).

28. Proestos, Y. *et al.* Present and future projections of habitat suitability of the Asian tiger mosquito , a vector of viral pathogens , from global climate simulation. *Philos. Trans. R. Soc. Lond. B. Biol. Sci.* **370**, (2015).

29. Khormi, H. M. & Kumar, L. Climate change and the potential global distribution of Aedes aegypti: spatial modelling using geographical information system and CLIMEX. *Geospatial Health* **8**, 405–15 (2014).

30. Capinha, C., Rocha, J. & Sousa, C. A. Macroclimate Determines the Global Range Limit of Aedes aegypti. *EcoHealth* **11**, 420–428 (2014).

31. Liu-Helmersson, J., Stenlund, H., Wilder-Smith, A. & Rocklöv, J. Vectorial capacity of Aedes aegypti: effects of temperature and implications for global dengue epidemic potential. *PloS One* **9**, e89783 (2014).
