# Supplementary material for: Past and future spread of the arbovirus vectors Aedes aegypti and Aedes albopictus
Source: Nat Microbiol. 2019 Mar 4;4(5):854–63. doi: 10.1038/s41564-019-0376-y (PMC6522366; doi:10.1038/s41564-019-0376-y)
Supplement: Supplementary file 2 — Reporting Summary [file 41564_2019_376_MOESM2_ESM.pdf]

nature research

Corresponding author(s): Simon I Hay & Moritz U G Kraemer

Reporting Summary

Nature Research wishes to improve the reproducibility of the work that we publish. This form provides structure for consistency and transparency in reporting. For further information on Nature Research policies, see Authors & Referees and the Editorial Policy Checklist.

Statistical parameters

When statistical analyses are reported, confirm that the following items are present in the relevant location (e.g. figure legend, table legend, main text, or Methods section).

n/a	Confirmed	
☐	☒	The exact sample size (n) for each experimental group/condition, given as a discrete number and unit of measurement
☐	☒	An indication of whether measurements were taken from distinct samples or whether the same sample was measured repeatedly
☐	☒	The statistical test(s) used AND whether they are one- or two-sided<br>*Only common tests should be described solely by name; describe more complex techniques in the Methods section.*
☐	☒	A description of all covariates tested
☐	☒	A description of any assumptions or corrections, such as tests of normality and adjustment for multiple comparisons
☐	☒	A full description of the statistics including central tendency (e.g. means) or other basic estimates (e.g. regression coefficient) AND variation (e.g. standard deviation) or associated estimates of uncertainty (e.g. confidence intervals)
☐	☒	For null hypothesis testing, the test statistic (e.g. F, t, r) with confidence intervals, effect sizes, degrees of freedom and P value noted<br>*Give P values as exact values whenever suitable.*
☒	☐	For Bayesian analysis, information on the choice of priors and Markov chain Monte Carlo settings
☐	☒	For hierarchical and complex designs, identification of the appropriate level for tests and full reporting of outcomes
☐	☒	Estimates of effect sizes (e.g. Cohen's d, Pearson's r), indicating how they were calculated
☐	☒	Clearly defined error bars<br>*State explicitly what error bars represent (e.g. SD, SE, CI)*

Our web collection on statistics for biologists may be useful.

Software and code

Policy information about availability of computer code

Data collection	Data collection and cleaning was performed in R, statistical software version 3.5.1 using standard R packages such as tidyverse: https://www.tidyverse.org/
Data analysis	Statistical analysis and plotting of results was performed in R, statistical software version 3.5.1

For manuscripts utilizing custom algorithms or software that are central to the research but not yet described in published literature, software must be made available to editors/reviewers upon request. We strongly encourage code deposition in a community repository (e.g. GitHub). See the Nature Research guidelines for submitting code & software for further information.

Data

Policy information about availability of data

All manuscripts must include a data availability statement. This statement should provide the following information, where applicable:

- Accession codes, unique identifiers, or web links for publicly available datasets
- A list of figures that have associated raw data
- A description of any restrictions on data availability

Data is available at: https://datadryad.org/resource/doi:10.5061/dryad.47v3c

Field-specific reporting

Please select the best fit for your research. If you are not sure, read the appropriate sections before making your selection.

☐ Life sciences ☐ Behavioural & social sciences ☒ Ecological, evolutionary & environmental sciences

For a reference copy of the document with all sections, see nature.com/authors/policies/ReportingSummary-flat.pdf

Ecological, evolutionary & environmental sciences study design

All studies must disclose on these points even when the disclosure is negative.

Study description	We used longitudinal data on mosquito occurrence to model the patterns spread in the USA and Europe using a generalised additive model. We further conducted a global analysis of potential spread using environmental data and a machine learning algorithm (Boosted Regression Tree) to map the potential global distribution of Aedes aegypti and Ae. albopictus mosquitoes.
Research sample	We conducted our analysis for the medically important Ae. aegypti and Ae. albopictus mosquitoes.
Sampling strategy	Sampling strategy is heterogenous and varies by country. Some of the samples are collected in response to nuisance reports by citizens. Others are collected routinely by efforts coordinated by the European Centers for Disease Control and Prevention (ECDC) or the US Government.
Data collection	We used secondary data collected by entomologists in many parts of the world. High resolution data from Europe and the United States are longitudinal. Data from other locations in the world are used from a previous study: https://www.nature.com/articles/sdata201535
Timing and spatial scale	Data were collected between 1980 - 2017 at highest spatial resolution possible (i.e., 5km by 5km pixel level or administrative unit level 2)
Data exclusions	No data were excluded prior to analysis
Reproducibility	We have uploaded all data and sample code to reproduce our findings.
Randomization	No randomization was performed. We did perform randomization within our statistical analysis leaving out mosquito observations.
Blinding	Not applicable

Did the study involve field work? ☐ Yes ☒ No

Reporting for specific materials, systems and methods

Materials & experimental systems

n/a	Involved in the study	
☒	☐	Unique biological materials
☒	☐	Antibodies
☒	☐	Eukaryotic cell lines
☒	☐	Palaeontology
☒	☐	Animals and other organisms
☒	☐	Human research participants

Methods

n/a	Involved in the study	
☒	☐	ChIP-seq
☒	☐	Flow cytometry
☒	☐	MRI-based neuroimaging
